# Gene expression signatures in childhood acute leukemias are largely unique and distinct from those of normal tissues and other malignancies

**DOI:** 10.1186/1755-8794-3-6

**Published:** 2010-03-08

**Authors:** Anna Andersson, Patrik Edén, Tor Olofsson, Thoas Fioretos

**Affiliations:** 1 Section of Clinical Genetics, Department of Laboratory Medicine, Lund University Hospital, Lund, Sweden; 2 Department of Complex System Division, Theoretical Physics, Lund University, Lund, Sweden; 3 Department of Hematology & Transfusion Medicine, Lund University, Lund, Sweden

## Abstract

**Background:**

Childhood leukemia is characterized by the presence of balanced chromosomal translocations or by other structural or numerical chromosomal changes. It is well know that leukemias with specific molecular abnormalities display profoundly different global gene expression profiles. However, it is largely unknown whether such subtype-specific leukemic signatures are unique or if they are active also in non-hematopoietic normal tissues or in other human cancer types.

**Methods:**

Using gene set enrichment analysis, we systematically explored whether the transcriptional programs in childhood acute lymphoblastic leukemia (ALL) and myeloid leukemia (AML) were significantly similar to those in different flow-sorted subpopulations of normal hematopoietic cells (n = 8), normal non-hematopoietic tissues (n = 22) or human cancer tissues (n = 13).

**Results:**

This study revealed that e.g., the t(12;21) [*ETV6-RUNX1*] subtype of ALL and the t(15;17) [*PML-RARA*] subtype of AML had transcriptional programs similar to those in normal Pro-B cells and promyelocytes, respectively. Moreover, the 11q23/*MLL *subtype of ALL showed similarities with non-hematopoietic tissues. Strikingly however, most of the transcriptional programs in the other leukemic subtypes lacked significant similarity to ~100 gene sets derived from normal and malignant tissues.

**Conclusions:**

This study demonstrates, for the first time, that the expression profiles of childhood leukemia are largely unique, with limited similarities to transcriptional programs active in normal hematopoietic cells, non-hematopoietic normal tissues or the most common forms of human cancer. In addition to providing important pathogenetic insights, these findings should facilitate the identification of candidate genes or transcriptional programs that can be used as unique targets in leukemia.

## Background

Genome wide analyses of human cancer have shown that genetic and epigenetic changes lead to deregulated cellular gene expression patterns. The aberrant transcriptional states of cancer cells are likely to consist of several transcriptional programs/modules that are important in the initiation and/or progression of malignancies. Recent work has successfully used deregulated gene expression profiles to classify different types of cancer and, in some cases, has led to the identification of new tumor subtypes [1-5]. However, forming biologically meaningful conclusions from the vast amount of genomic data has proven more challenging than first anticipated [6].

Childhood leukemia is the most common pediatric malignancy. It is typically characterized by balanced chromosomal translocations that form oncogenic fusion genes or by other structural or numerical chromosomal changes. For example, acute lymphoblastic leukemia (ALL) is characterized by the following specific alterations: t(12;21)(p13;q22) [*ETV6/RUNX1*], high hyperdiploidy (HeH, >50 chromosomes), t(1;19)(q23;p13) [*TCF3/PBX1*], and t(9;22)(q34;q22) [*BCR/ABL1*], whereas acute myeloid leukemia (AML) is characterized by t(8;21)(q22;q22) [*RUNX1/RUNX1T1*], t(15;17)(q22;q21) [*PML/RARA*], and inv(16)(p13q22) [*CBFB/MYH11*]. Furthermore, 11q23/*MLL *rearrangements are present in both childhood ALL and AML. The presence of these genetic abnormalities provides important diagnostic and prognostic information in a clinical setting [7,8]. The oncogenic properties of several fusion genes have been studied in different mouse models and their transforming capacities have been firmly established [9]. However, it is not understood how specific fusion genes alter the normal transcriptional programs that tightly regulate self-renewal and differentiation from stem cells to mature blood cells. Although global genomic studies have shown that various human leukemia subtypes display profoundly different gene expression profiles [2,4,5,10-12], we still lack important information about the biological and pathogenetic impact of these deregulated transcriptional programs.

We have previously shown that the interpretation of aberrant transcriptional signatures in childhood acute lymphoblastic leukemia (ALL) can be improved by including normal hematopoietic subpopulations [10]. For example, we have demonstrated that relatively few of the top differentially expressed genes in the different genetic subtypes of childhood leukemia were correlated with hematopoietic lineages, i.e., myeloid-, lymphoid, or T-cell lineages. Instead, we found that most of the genes were either highly expressed in immature normal hematopoietic cells or displayed a seemingly 'ectopic expression' restricted to the leukemic cells only. In the current study, we have extended our earlier observations by systematically exploring whether the transcriptional programs in various genetic subtypes of childhood ALL and acute myeloid leukemia (AML) show any significant similarities to those present in an enlarged set of flow-sorted normal hematopoietic cells, a large number of normal non-hematopoietic tissues, or different types of human cancer. We report that, with few exceptions, the transcriptional programs of most childhood acute leukemia subtypes display limited similarities to ~100 gene sets representing transcriptional programs/modules active in normal and malignant tissues.

## Results and Discussion

Gene expression profiling has been extensively used in the past to identify differentially expressed genes in childhood leukemia [2,4,5,10]. However, it remains challenging to make biological meaningful conclusions from such gene lists. Typically, investigators analyze these gene lists by different gene ontology or pathway analysis tools that may identify certain gene ontology categories or pathways that are perturbed, thus facilitating biological interpretation. In the past, we have used similar tools and identified several deregulated gene ontology terms being deregulated in leukemia. For example, leukemias characterized by the t(1;19) *TCF3/PBX1 *fusion gene showed a significant enrichment of cell proliferation and cell cycle genes, possibly reflecting the aggressive phenotype of this leukemia, which, before intensive treatment protocols were introduced often presented with adverse clinical symptoms such as central nervous system disease and with a high risk of relapse [10,13]. In addition, we have previously shown that the inclusion of normal hematopoietic cells can improve the interpretation of gene expression patterns in leukemia [10], by highlighting genes that display a shared or unique expression pattern in the various normal hematopoietic cells and the different genetic subtypes of leukemia.

In the present study, we undertook another approach in trying to understand the biological meaning of the vast number of genes that are differentially expressed in childhood leukemia. Using gene set enrichment analysis (GSEA) [14], which provides a powerful tool to ascertain whether a given gene set is significantly enriched in a list of genes ranked by their correlation with a phenotype of interest [14,15], we systematically explored whether differentially expressed genes in the various subtypes of childhood leukemia display enrichment of genes found differentially expressed in different normal and malignant tissues. The rationale behind this approach is that gene sets that occur in more than one condition may provide pathogenetic clues for understanding the nature of the aberrant transcriptional programs present in different genetic subtypes of childhood leukemia. Moreover, by identifying genes or transcriptional programs displaying a confined expression only to the leukemic cells and with absent expression in normal hematopoietic cells, new candidates for targeted treatment may be identified. Finally, the results may give rise to new ideas for treating leukemia by identifying other tumor types showing similar types of aberrant transcriptional programs.

**Figure 1:**
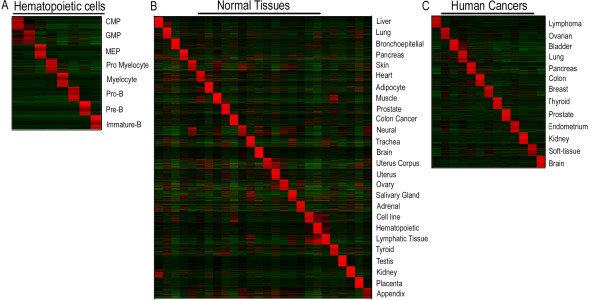
**Heat map of the top 100 upregulated genes from each gene set used in the GSEA analyses**. Genes are located vertically and samples horizontally in the order given to the left in figures A-C. Each sample is represented by the mean of the gene expression value of the samples within that subgroup. The figures shows the 100 most highly expressed genes from each of the different gene sets used in the GSEA analyses from A) the flow sorted hematopoietic cells, B) the normal tissues, and C) the different human cancers. Red indicates relative upregulation and green relative downregulation.

To generate the different gene sets, we used flow-sorting to isolate eight subpopulations of hematopoietic cells of different lineage and maturation from normal donors and established their gene expression patterns. In addition, we downloaded raw gene expression data from normal non-hematopoietic tissues [16] and various types of common human malignancies https://expo.intgen.org/geo/dataDownload.do. Gene sets were generated within each data set from the flow sorted normal hematopoietic cells, the normal tissue data sets as well as from the different malignancies. This database of gene sets was then used to search for similarities towards the gene expression patterns that characterize the various molecular subtypes of childhood leukemia. In total, close to 100 transcriptional signatures or 'gene sets' derived from eight different flow-sorted normal hematopoietic cell subpopulations, 22 normal non-hematopoietic tissues, and 13 of the most common human cancer types (Figure 1) were investigated for enrichment in the gene expression profiles obtained from different genetic subtypes of childhood ALL and AML.

**Figure 2:**
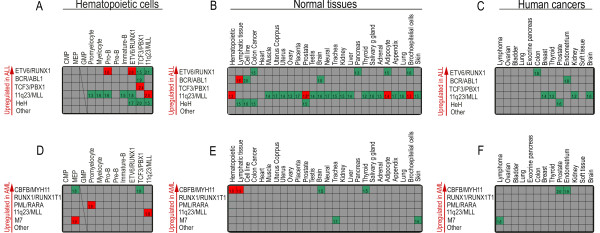
**Summary of the enrichments found in childhood ALL and AML when analyzed against gene sets derived from a large set of normal hematopoietic cells, normal tissues and different types of human cancers using GSEA**. Only enrichments found among the upregulated genes in the various leukemia subtypes and the different gene sets are shown. A-C depict enrichments found in ALL and D-F in AML. A red colored square indicate a significant enrichment among genes being upregulated in a genetic subtype with genes contained within a gene set being upregulated in a specific tissue. A green colored square indicates a significant enrichment among genes being upregulated in a genetic subtype with genes contained within a gene set that is downregulated in a specific tissue. The number in the squares shows the normalized enrichment score, which can be used to compare the results across gene sets. Only scores for significant enrichments as determined by FDR are shown in the figure. A and D) genes contained in genes sets from normal flow sorted hematopoietic cells. Also included, as positive controls, are gene sets from previously generated gene expression data of pediatric ALL, B and E) genes contained in genes sets from a large set of different normal human tissues, and C and F) genes contained in genes sets from different human cancers.

**Figure 3:**
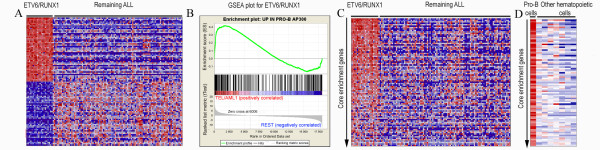
**GSEA reveals similarities between genes upregulated in *ETV6/RUNX1 *positive ALL and those upregulated in normal Pro-B cells**. A) Heat map generated within GSEA of the top ranked 50 up- and downregulated genes in *ETV6/RUNX1*-positive leukemia as compared to the remaining ALLs. B) Enrichment plot showing the enrichment of genes upregulated in Pro-B cells among the top ranked upregulated genes in ALLs with *ETV6/RUNX1*. The lower portion of the figure shows the rank ordered genes for *ETV6/RUNX1*-positive ALL when compared to other ALL, with genes being highly expressed to the far left and downregulated genes to the right. Each black line represents a hit from the gene set being investigated in the rank ordered gene list. The more hits among the top up- or downregulated genes, the more likely you are to get a significant gene set enrichment score. The upper part of the figure shows the enrichment score, which is calculated by walking down the ranked ordered gene list and increasing the score when a gene from the rank ordered gene list is in the gene set and decreasing it when it is not. C) Heat map of the core enrichment genes in the *ETV6/RUNX1 *positive ALL and the remaining ALLs. D) Heat map of the same core enrichment genes in the normal flow sorted hematopoietic cells. The order of the core enrichment genes is the same as in C. From figure C and D, the similarity of the *ETV6/RUNX1 *and the Pro-B cell gene expression profiles can be easily appreciated and clearly shows that there are transcriptional programs that are shared between this specific leukemia and its normal counterpart.

First, we investigated whether our newly generated gene sets derived from eight flow-sorted subpopulations of normal hematopoietic cells were enriched within the gene expression profiles of the various genetic subtypes of childhood ALL/AML. This analysis would indicate whether transcriptional programs are shared between normal hematopoietic cells and leukemic blasts. The enrichment of a particular gene set could also indirectly provide clues as to the 'cellular origin' of a leukemia by displaying enrichment of a gene set from a particular subpopulation of normal hematopoietic cells in the gene expression profile of a specific leukemic subtype. Among the childhood ALL subtypes, *ETV6/RUNX1 *was the only subtype where gene sets obtained from normal hematopoietic cells was enriched: namely with genes that were highly expressed in normal Pro-B cells (Figure 2 and 3; additional file 1). This is consistent with a recent study that demonstrated that the leukemia propagating cells in *ETV6/RUNX1*-positive ALL, likely are restricted to CD34^+^CD38^-/low^/CD19^+ ^cells, which display a mixture of gene expression profiles characteristic of normal Pro-B cells and hematopoietic stem cells [17]. Surprisingly, no other ALL subtype displayed upregulated genes that significantly overlapped with gene sets derived from the normal hematopoietic cell subpopulations. This suggests that the gene expression profiles of these ALL subtypes are derived from a more immature hematopoietic subpopulation or contain genes that are ectopically expressed specifically in leukemia, as we have previously described [10]. If the latter case is true, it would suggest that the primary genetic change in leukemia, typically a fusion oncogene, elicits a transcriptional program that is unique to each genetic subtype of leukemia and hence, is not used by normal hematopoietic cells. Alternatively, and perhaps more likely, the gene expression profiles reflect a combination of several transcriptional programs: 1) those active in the cell targeted by the primary genetic change, and 2) those that become deregulated as a consequence of the specific genetic changes that occur during leukemogenesis.

ALL subtypes with 11q23/*MLL *rearrangements showed an inverse correlation to the gene expression profiles of normal Pro-B cells, where genes highly expressed in the leukemia were downregulated in normal Pro-B cells, and vice versa (Figure 2 and additional file 2). The biological significance of these correlations is presently unclear, but could indicate that the transcriptional programs governing B-cell regulation is perturbed in this leukemia subtype. As expected, a high correlation was obtained for gene sets derived from our previous cDNA microarray analysis of *ETV6/RUNX1*, *TCF3/PBX1*, and 11q23/*MLL*-positive ALL subtypes (Figure 2, additional file 2) [10]. Among the downregulated genes, the *TCF3/PBX1 *positive subtype of ALL was the only subtype showing enrichment among the normal hematopoietic cells, namely with genes downregulated in normal pro-B cells and genes downregulated in MEP cells (additional file 2).

Among the childhood AML subtypes, we found that the *PML/RARA *subtype, which contains a fusion gene that is tightly associated with promyelocytic leukemia, not unexpectedly displayed upregulated genes that were highly correlated to the upregulated genes in normal promyelocytes (Figure 2, additional file 3). This fusion gene, when introduced into human hematopoietic stem cells (HSC) rapidly induces differentiation of the HSC into promyelocytes and then blocks differentiation [18]. In addition, among the downregulated genes, enrichment was observed among genes being downregulated in normal pro-B cells (additional file 2). We also found that the AML subtype, M7, which is associated with a dismal prognosis[19], displayed genome-wide similarities to normal MEP cells (Figure 2, additional file 4, and additional file 5). This may reflect the cellular level of transformation and/or maturation arrest of this subtype of leukemia [20]. These analyses clearly demonstrated the power of GSEA to capture important similarities between the transcriptional programs of leukemic cells and those of normal hematopoietic cell subpopulations.

**Figure 4:**
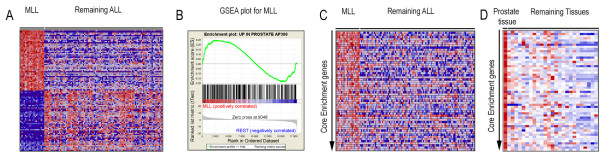
**GSEA reveals similarities between the genes being upregulated in ALL with 11q23/*MLL *and those upregulated in normal prostate tissues**. A) Heat map of the top ranked 50 up- and downregulated genes in 11q23/*MLL*-positive ALL. B) Enrichment plot showing the enrichment of genes upregulated in normal prostate tissue among the upregulated genes in ALLs with 11q23/*MLL*. For an explanation about the enrichment plot, see the legend to Figure 3B. C) Heat map of the core enrichment genes in the ALL data set. D) Heat map of the same core enrichment genes in the normal tissue data, the order of the core enrichment genes are the same as in C. When comparing figures C and D, the similarity of the 11q23/*MLL *and the prostate tissue gene expression profiles can be easily appreciated, indicating a shared transcriptional program.

Next, we investigated whether gene sets derived from a large number of normal non-hematopoietic tissues were enriched within the gene expression profiles of childhood ALL and AML. For this purpose, we downloaded gene expression data from a large number of normal human tissues [16] and generated 52 gene sets corresponding to 26 tissues. This analysis revealed that, among the upregulated genes, only the 11q23/*MLL*-positive subtype of ALL showed significant similarities with other normal tissues, where gene sets from prostate, adipocytes, and bronchoepithelial cells were enriched among the upregulated genes in such ALLs (Figure 2, and 4, additional file 6, additional file 7, and additional file 8). Notably, this was not evident in childhood AML with the 11q23/*MLL *abnormality. When investigating the overlap between the downregulated genes, again, the gene set derived from bronchoepithelial cells were enriched among the 11q23/*MLL *positive ALL (additional file 2). Also, the *TCF3*/*PBX1 *positive subtype of ALL shared similarities with several normal tissues with regard to their downregulated genes (additional file 2). The remaining enrichments observed were among genes upregulated in the various genetic subtypes of childhood ALL and AML and those downregulated in several other non-hematopoietic tissues (Figure 2).

Finally, we investigated whether gene sets derived from several different human malignancies were enriched within the gene expression profiles of childhood leukemia subtypes. In this analysis, 13 different human cancers, totaling 75 individual tumor samples were imported from the expO database https://expo.intgen.org/geo/dataDownload.do. The 26 different gene set generated from this data set included the most common human cancers; breast, colon, prostate, and lung cancer. Interestingly, with respect to the upregulated genes, none of the gene sets derived from the different human cancer types were enriched within the gene expression profiles of the various leukemic subtypes (Figure 2). This finding indicates that the perturbed transcriptional profiles of childhood leukemia are different from those of other human cancer types.

A striking observation in the present study was that the majority of the enrichments detected in the GSEA analyses were negative correlations (Figure 2). That is, several genes that were highly expressed in the various genetic subtypes of leukemia were downregulated in normal and malignant tissues. This further supports our hypothesis that the gene expression programs that are transcriptionally active in the various subtypes of childhood leukemia are unique to the leukemia and show limited similarity to other tissues and human cancers. Interestingly, childhood ALL and AML differed substantially in the number of enrichments found by GSEA; we identified 100 enrichments in ALL and only 26 in AML. This may indicate that childhood AML activate transcriptional modules that are more disease-specific than those activated in ALL.

We are aware of only one previous study in which a genome-wide comparison was performed between genes being differentially expressed in leukemia and in normal tissues [21]. Using 'gene per gene' comparison, Lotem et al, 2005 [21] reported that several ALL subtypes overexpressed genes that were active in normal testis or neural tissues. In contrast, we used GSEA that ascertains whether several genes contained within a gene set are significantly enriched within the gene expression profiles of the various genetic subtypes of childhood leukemia. Although the two approaches are different, our results are partly consistent with those of Lotem et al., 2005 [21]. For example, in a 'gene per gene' comparison we also find that the *ETV6/RUNX1 *ALL subtype overexpress 45 genes that are highly expressed in normal testis, but with GSEA, this did not reach statistical significance.

## Conclusions

Our findings suggest that childhood leukemia display altered expression profiles that are largely unique to the leukemic cells, with only limited similarities to those in other tissues or in the most common forms of human cancer. Further dissection of these perturbed transcriptional programs should provide additional important pathogenetic insights and may help promoting the development of agents that target key mediators of the aberrant transcriptional programs uniquely expressed in the various genetic subtypes of childhood leukemia.

## Methods

### Expression profiling

Eight flow-sorted subpopulations, obtained after informed consent from healthy adult donors through the Department of Hematology, Lund University Hospital, Sweden, were analyzed. The study was reviewed and approved by the Research Ethic Committee of Lund University, Sweden. These eight subpopulations included: 1) common myeloid progenitor (CMP) cells, 2) granulocyte/macrophage progenitor (GMP) cells, 3) megakaryocyte/erythrocyte progenitor (MEP) cells, 4) myeloid cells enriched for promyelocytes, 5) myeloid cells enriched for myelocytes, 6) pro-B cells, 7) pre-B cells, and 8) immature-B cells. The immunophenotype, and gating strategy are shown in additional file 9. To obtain sufficient amount of RNA from each subpopulation, cells from one to six separate sorting experiments were pooled. Two independently pooled samples were subsequently hybridized onto Human Genome U133 Plus 2.0 microarrays (Affymetrix Inc, Santa Clara, CA). Data have been deposited in Gene Expression Omnibus (GSE19599). For detailed methods information, see additional file 10 (available on the BMC Medical Genomics website).

### External data sets

Raw expression data from Ross et al., 2003 and 2004 [4,5] on childhood ALL and AML were downloaded from http://www.stjuderesearch.org. Data composition can be viewed in Supplemental Methods (additional file 10). Normal tissue and human cancer expression data were retrieved from two external data sets. The first [16] consisted of 79 human tissues hybridized in duplicate. Samples from the same types of tissue were combined, and a mean gene expression value was calculated and used for subsequent analyses (additional file 11). The second set, consisting of human tumors, was obtained from the expO database https://expo.intgen.org/geo/dataDownload.do. Cel-files from solid tumors of different types and origin were grouped based on the site of the primary tumor. In total, 75 cases from 13 different tumor sites (2-9 cases per site; mean 5.7 cases per site), including breast, colon, prostate, and lung, were included (additional file 12). After normalization, samples of the same type were combined and the mean expression value was used for subsequent analysis (see below).

### Data normalization and gene set enrichment analysis

Normalization of all data sets was performed using the gcRMA [22] algorithm in the R statistical language as part of the Bioconductor bioinformatics software [23]. For the data sets that were used to produce gene sets (see below), log2 values corresponding to a gene expression value below 10 were set to a value of 10. All data were mean-centered and subjected to a variation filter, discarding all probe sets with a standard deviation below 0.5. In the data sets from normal flow-sorted hematopoietic cells, non-hematopoietic tissues, and human cancer tissues, differentially expressed genes were identified by ranking genes with *t*-statistics and a false discovery rate (FDR). The mean value and the within group variance for each tissue type was used for calculating the *t*-statistics. For each data set, the ranked gene lists were generated by comparing the group of interest to the remaining samples in that data set.

To search for enrichment of specific 'transcriptional programs' (herein defined as 'gene sets') within the different subtypes of childhood leukemia, we used GSEA [14,15]. GSEA is a computational method that ascertains whether a given gene set (in our case derived from the different normal and malignant tissues) is significantly enriched in a list of genes ranked by their correlation with a phenotype of interest (herein the various genetic subtypes of childhood leukemia).

To generate the different gene sets, a list of genes that corresponded to each tissue of interest was truncated at a FDR of 1% or alternatively, for very low FDR values, the list comprised approximately 300 unique genes. From each gene list, two gene sets were generated, one for the upregulated and one for the downregulated genes. In total, 99 gene sets were produced from the normal hematopoietic cells, the normal tissues, and the different human cancer samples. The top 100 genes in each of the gene sets derived from the upregulated genes in the three data sets, are depicted in Figure 1. As a control, we included six gene sets from our previously generated lists of genes that were differentially expressed in childhood ALL with *ETV6/RUNX1*, *TCF3/PBX1*, or 11q23/*MLL*-rearrangements [10]. Within GSEA [14], enrichment analysis was performed using the option of collapsing probe sets in the gene expression data matrix to gene symbols. The ALL and AML data sets were not subjected to a variation filter and probes with multiple hits in the genome were removed. To produce the ranked gene lists for each genetic subtype of ALL and AML within GSEA, we used *t*-statistics and 2000 phenotype permutations to generate the normalized enrichment score. Only gene sets that contained more than 15 and below 500 genes were considered, resulting in the exclusion of three gene sets that included genes up- or downregulated in GMP cells (14 and 0 genes, respectively) and genes downregulated in CMP cells (3 genes). Correlations in GSEA are measured by a Normalized Expression Score, which is the Kolmogorov-Smirnov running sum [24,25], divided by the mean of the corresponding result for permuted datasets. The permutation results are also used to assign a FDR for the enrichment. A FDR of 25% (indicating that the enrichment is valid 3 out of 4 times) or below was considered significant.

## Competing interests

The authors declare that they have no competing interests.

## Authors' contributions

AA and TF designed the research and wrote the manuscript. AA, PE, TO and TF performed the research and analyzed the data. All authors have read and approved the final manuscript.

## Pre-publication history

The pre-publication history for this paper can be accessed here:

http://www.biomedcentral.com/1755-8794/3/6/prepub

## Supplementary Material

Additional file 1 **Core enrichment genes in pediatric ALL with *ETV6/RUNX1 *when compared to genes being upregulated in normal Pro-B cells**. Table of the core enrichment genes, their rank and statistics from the gene set enrichment analysis. Click here for file

Additional file 2 **A Summary of the enrichments found among the downregulated genes in childhood ALL and AML using GSEA**. Figure showing the enrichment scores among the dowregulated genes in the various genetic subtypes of ALL and AML. Click here for file

Additional file 3 **GSEA reveals similarities between genes upregulated in pediatric APL with *PML/RARA *and those upregulated in normal promyelocytes**. Heat maps and enrichment plots of the comparison of normal promyelocytes and APL. Click here for file

Additional file 4 **GSEA reveals similarities between genes being upregulated in pediatric AML with AML M7 and those upregulated in normal MEP cells**. Heat maps and enrichment plots of the comparison of normal MEP cells and AML M7. Click here for file

Additional file 5 **Core enrichment genes in pediatric AML M7 when compared to genes being upregulated in normal flow sorted MEP cells**. Table of the core enrichment genes, their rank and statistics from the gene set enrichment analysis. Click here for file

Additional file 6 **Core enrichment genes in pediatric ALL with 11q23/*MLL *when compared to genes being upregulated in normal bronchoepitelial cells**. Table of the core enrichment genes, their rank and statistics from the gene set enrichment analysis. Click here for file

Additional file 7 **Core enrichment genes in pediatric ALL with 11q23/*MLL *when compared to genes being upregulated in normal adipocytes**. Table of the core enrichment genes, their rank and statistics from the gene set enrichment analysis. Click here for file

Additional file 8 **Core enrichment genes in pediatric ALL with *MLL *when compared to genes being upregulated in normal tissue derived from prostate**. Table of the core enrichment genes, their rank and statistics from the gene set enrichment analysis. Click here for file

Additional file 9 **Isolation strategy of the different subpopulations analyzed for gene expression**. Example of the isolation strategy of the hematopoietic subpopulations. Click here for file

Additional file 10 **Supporting Methods**. Contains additional methods information. Click here for file

Additional file 11 **The normal tissue data set by Su el al., 2005 and the annotations used for producing the gene sets**. Cel files and annotations for the data set by Su et al., 2005. Click here for file

Additional file 12 **The solid tumor data set from the exp*O *database**. Description of the analyzed tumors. Click here for file
