# Supplementary material for: Gene expression signatures in childhood acute leukemias are largely unique and distinct from those of normal tissues and other malignancies
Source: BMC Med Genomics. 2010 Mar 8;3:6. doi: 10.1186/1755-8794-3-6 (PMC2845086; doi:10.1186/1755-8794-3-6)
Supplement: Additional file 1 — Core enrichment genes in pediatric ALL with ETV6/RUNX1 when compared to genes being upregulated in normal Pro-B cells. Table of the core enrichment genes, their rank and statistics from the gene set enrichment analysis. [file 1755-8794-3-6-S1.DOC]

**Additional file 1**. Core enrichment genes in pediatric ALL with *ETV6/RUNX1* when compared to genes being upregulated in normal Pro-B cells.

| *GENE SYMBOL* | *GENE TITLE* | *RANK IN GENE LIST* | *RANK METRIC SCORE* | *RUNNING ES* | *CORE ENRICHMENT* |
| --- | --- | --- | --- | --- | --- |
| TNFRSF21 | tumor necrosis factor receptor superfamily, member 21 | 3 | 18.46161 | 0.020736 | Yes |
| TERF2 | telomeric repeat binding factor 2 | 7 | 16.42542 | 0.039166 | Yes |
| RAG1 | recombination activating gene 1 | 15 | 13.34176 | 0.05388 | Yes |
| GNG11 | guanine nucleotide binding protein (G protein), gamma 11 | 34 | 11.4311 | 0.065812 | Yes |
| ZNF423 | zinc finger protein 423 | 40 | 11.12773 | 0.078132 | Yes |
| SMAD1 | SMAD, mothers against DPP homolog 1 (Drosophila) | 42 | 11.06233 | 0.090602 | Yes |
| STK32B | serine/threonine kinase 32B | 47 | 10.70216 | 0.102495 | Yes |
| TSPAN7 | tetraspanin 7 | 71 | 9.446804 | 0.1119 | Yes |
| KHDRBS3 | KH domain containing, RNA binding, signal transduction associated 3 | 76 | 9.065667 | 0.121941 | Yes |
| DBN1 | drebrin 1 | 84 | 8.805232 | 0.131518 | Yes |
| GAB1 | GRB2-associated binding protein 1 | 101 | 8.166324 | 0.139866 | Yes |
| FBXW7 | F-box and WD-40 domain protein 7 (archipelago homolog, Drosophila) | 110 | 7.829423 | 0.148282 | Yes |
| SMARCA4 | SWI/SNF related, matrix associated, actin dependent regulator of chromatin, subfamily a, member 4 | 114 | 7.745071 | 0.156884 | Yes |
| NARFL | nuclear prelamin A recognition factor-like | 122 | 7.575204 | 0.165068 | Yes |
| COL5A1 | collagen, type V, alpha 1 | 137 | 7.304749 | 0.172553 | Yes |
| SHANK3 | SH3 and multiple ankyrin repeat domains 3 | 146 | 7.092342 | 0.180135 | Yes |
| LOC152485 | - | 149 | 7.017969 | 0.187969 | Yes |
| VPREB3 | pre-B lymphocyte gene 3 | 158 | 6.935213 | 0.195372 | Yes |
| PSD3 | pleckstrin and Sec7 domain containing 3 | 174 | 6.765265 | 0.20219 | Yes |
| FAM134B | Null | 181 | 6.661096 | 0.209396 | Yes |
| ZNF608 | zinc finger protein 608 | 185 | 6.594449 | 0.216694 | Yes |
| FHIT | fragile histidine triad gene | 189 | 6.541882 | 0.223933 | Yes |
| ABCG2 | ATP-binding cassette, sub-family G (WHITE), member 2 | 196 | 6.496782 | 0.230953 | Yes |
| CTA-221G9.4 | - | 200 | 6.454754 | 0.238093 | Yes |
| CYGB | Cytoglobin | 203 | 6.396119 | 0.245223 | Yes |
| MYLK | myosin, light chain kinase | 207 | 6.343107 | 0.252237 | Yes |
| YEATS2 | YEATS domain containing 2 | 208 | 6.330764 | 0.259405 | Yes |
| SPANXC | SPANX family, member C | 210 | 6.315964 | 0.266501 | Yes |
| MME | membrane metallo-endopeptidase (neutral endopeptidase, enkephalinase) | 213 | 6.288671 | 0.273509 | Yes |
| CRMP1 | collapsin response mediator protein 1 | 214 | 6.276919 | 0.280617 | Yes |
| ZMIZ1 | zinc finger, MIZ-type containing 1 | 224 | 6.126929 | 0.287049 | Yes |
| CTGF | connective tissue growth factor | 226 | 6.105082 | 0.293905 | Yes |
| BEST3 | bestrophin 3 | 240 | 5.970724 | 0.299936 | Yes |
| AXUD1 | AXIN1 up-regulated 1 | 272 | 5.679549 | 0.304626 | Yes |
| MLXIP | MLX interacting protein | 279 | 5.650275 | 0.310687 | Yes |
| SCARB1 | scavenger receptor class B, member 1 | 289 | 5.566916 | 0.316485 | Yes |
| LOC441108 | - | 291 | 5.544152 | 0.322707 | Yes |
| CMTM7 | CKLF-like MARVEL transmembrane domain containing 7 | 308 | 5.484983 | 0.328019 | Yes |
| RECK | reversion-inducing-cysteine-rich protein with kazal motifs | 320 | 5.422826 | 0.333542 | Yes |
| EFNA1 | ephrin-A1 | 379 | 4.994862 | 0.335941 | Yes |
| HPS4 | Hermansky-Pudlak syndrome 4 | 386 | 4.924486 | 0.34118 | Yes |
| SCMH1 | sex comb on midleg homolog 1 (Drosophila) | 410 | 4.792847 | 0.345315 | Yes |
| HIST1H2BF | histone cluster 1, H2bf | 431 | 4.672648 | 0.349483 | Yes |
| AKAP12 | A kinase (PRKA) anchor protein (gravin) 12 | 457 | 4.514734 | 0.353191 | Yes |
| IRX1 | iroquois homeobox protein 1 | 470 | 4.470406 | 0.357579 | Yes |
| ZNF117 | zinc finger protein 117 | 477 | 4.439207 | 0.362269 | Yes |
| MGC5370 | - | 491 | 4.34457 | 0.366459 | Yes |
| GABPB2 | GA binding protein transcription factor, beta subunit 2 | 500 | 4.282612 | 0.370859 | Yes |
| SIAH2 | seven in absentia homolog 2 (Drosophila) | 570 | 3.944813 | 0.371451 | Yes |
| WFS1 | Wolfram syndrome 1 (wolframin) | 582 | 3.914652 | 0.375266 | Yes |
| LOC283454 | - | 644 | 3.696545 | 0.376026 | Yes |
| CRIM1 | cysteine rich transmembrane BMP regulator 1 (chordin-like) | 697 | 3.55741 | 0.377134 | Yes |
| NPY | neuropeptide Y | 724 | 3.489701 | 0.379626 | Yes |
| IKZF1 | IKAROS family zinc finger 1 (Ikaros) | 755 | 3.409853 | 0.381802 | Yes |
| SPTBN1 | spectrin, beta, non-erythrocytic 1 | 784 | 3.322101 | 0.383992 | Yes |
| LOC646576 | - | 785 | 3.320741 | 0.387752 | Yes |
| DAGLB | Null | 788 | 3.312872 | 0.391391 | Yes |
| HIST1H2BH | histone cluster 1, H2bh | 832 | 3.213992 | 0.392615 | Yes |
| FAM80B | family with sequence similarity 80, member B | 860 | 3.151133 | 0.394667 | Yes |
| DNTT | deoxynucleotidyltransferase, terminal | 950 | 2.942477 | 0.393002 | Yes |
| LYPLA3 | lysophospholipase 3 (lysosomal phospholipase A2) | 978 | 2.902413 | 0.394772 | Yes |
| XPO7 | exportin 7 | 1089 | 2.725551 | 0.391681 | Yes |
| OAS3 | 2'-5'-oligoadenylate synthetase 3, 100kDa | 1111 | 2.699636 | 0.393559 | Yes |
| PPP2R3B | protein phosphatase 2 (formerly 2A), regulatory subunit B'', beta | 1166 | 2.613964 | 0.393487 | Yes |
| DVL3 | dishevelled, dsh homolog 3 (Drosophila) | 1183 | 2.589558 | 0.39552 | Yes |
| PXDN | peroxidasin homolog (Drosophila) | 1203 | 2.548867 | 0.39734 | Yes |
| HRK | harakiri, BCL2 interacting protein (contains only BH3 domain) | 1205 | 2.548201 | 0.400169 | Yes |
| ARPP-21 | - | 1222 | 2.532509 | 0.402138 | Yes |
| TSPAN9 | tetraspanin 9 | 1282 | 2.444969 | 0.401593 | Yes |
| RAD54L2 | RAD54-like 2 (S. cerevisiae) | 1318 | 2.408205 | 0.402355 | Yes |
| PPP2R2C | protein phosphatase 2 (formerly 2A), regulatory subunit B (PR 52), gamma isoform | 1349 | 2.376493 | 0.403361 | Yes |
| GNAS | GNAS complex locus | 1456 | 2.222974 | 0.399926 | Yes |
| SORBS3 | sorbin and SH3 domain containing 3 | 1474 | 2.203443 | 0.401467 | Yes |
| TOP2B | topoisomerase (DNA) II beta 180kDa | 1475 | 2.202478 | 0.403961 | Yes |
| HIST1H3H | histone cluster 1, H3h | 1476 | 2.201914 | 0.406454 | Yes |
| MYBL2 | v-myb myeloblastosis viral oncogene homolog (avian)-like 2 | 1490 | 2.183776 | 0.408197 | Yes |
| COBL | cordon-bleu homolog (mouse) | 1505 | 2.169517 | 0.409867 | Yes |
| HCFC1 | host cell factor C1 (VP16-accessory protein) | 1507 | 2.16763 | 0.412265 | Yes |
| ZNF85 | zinc finger protein 85 | 1576 | 2.084291 | 0.410807 | Yes |
| LIG4 | ligase IV, DNA, ATP-dependent | 1601 | 2.057925 | 0.41179 | Yes |
| DLG3 | discs, large homolog 3 (neuroendocrine-dlg, Drosophila) | 1648 | 2.015789 | 0.411489 | Yes |
| AHDC1 | AT hook, DNA binding motif, containing 1 | 1715 | 1.951692 | 0.409993 | Yes |
| PLXNA1 | plexin A1 | 1719 | 1.947582 | 0.41203 | Yes |
| CENPN | centromere protein N | 1720 | 1.946763 | 0.414234 | Yes |
| LOC339047 | - | 1753 | 1.907841 | 0.414598 | Yes |
| DUSP26 | dual specificity phosphatase 26 (putative) | 1823 | 1.845736 | 0.412813 | Yes |
| PAQR4 | progestin and adipoQ receptor family member IV | 1833 | 1.834226 | 0.414385 | Yes |
| E2F1 | E2F transcription factor 1 | 1853 | 1.816236 | 0.415374 | Yes |
| TYMS | thymidylate synthetase | 1886 | 1.786646 | 0.415601 | Yes |
