# Supplementary material for: Gene expression signatures in childhood acute leukemias are largely unique and distinct from those of normal tissues and other malignancies
Source: BMC Med Genomics. 2010 Mar 8;3:6. doi: 10.1186/1755-8794-3-6 (PMC2845086; doi:10.1186/1755-8794-3-6)
Supplement: Additional file 2 — A Summary of the enrichments found among the downregulated genes in childhood ALL and AML using GSEA. Figure showing the enrichment scores among the dowregulated genes in the various genetic subtypes of ALL and AML. [file 1755-8794-3-6-S2.DOC]

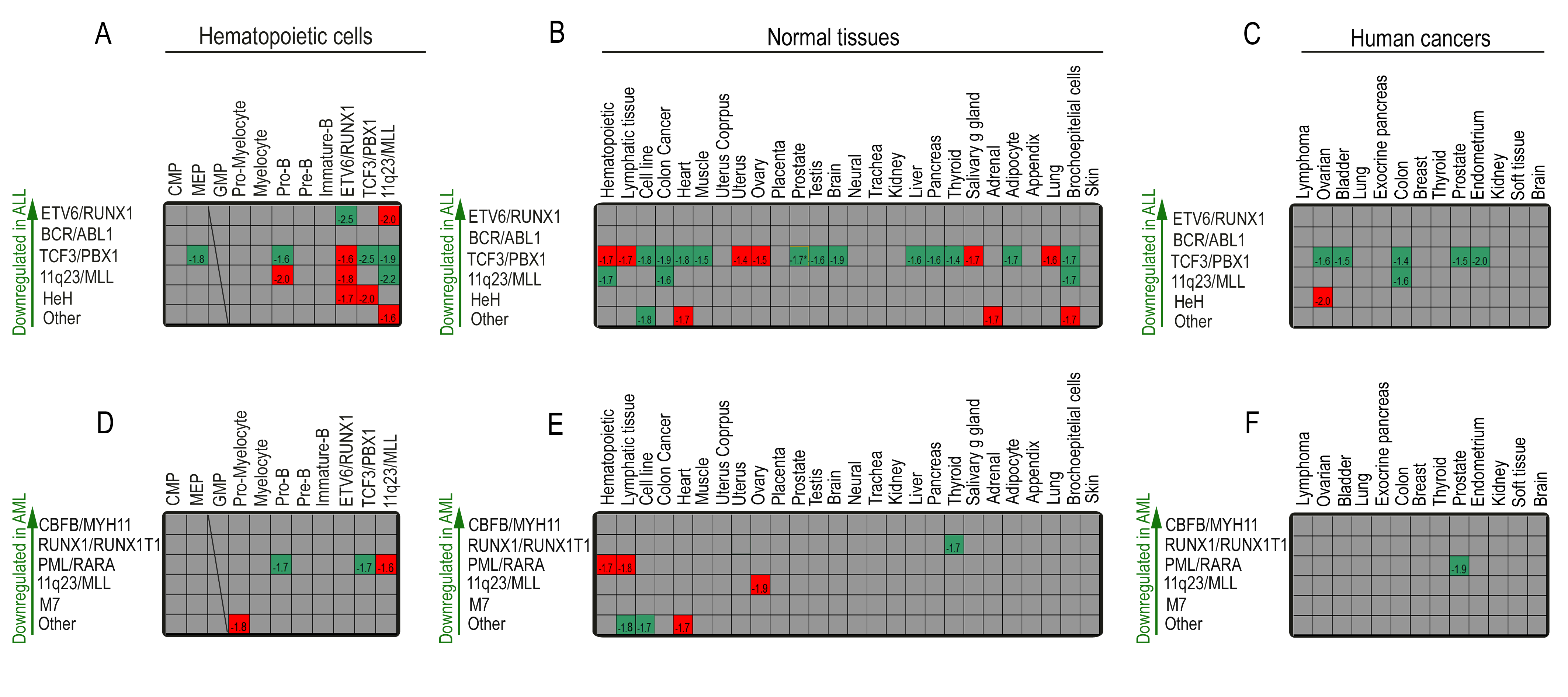


**Legend to Additional file 2**. Summary of the enrichments found in childhood ALL and AMLusing GSEA. Only enrichments among the downregulated genes in the different genetic

subtypes are shown. A-C depicts enrichments found in ALL and D-F in AML. A red

colored square indicate a significant enrichment in a leukemic subtype with genes

contained within a gene set being upregulated in a specific tissue. A green colored square

indicate a significant enrichment in a leukemic subtype with genes contained within a

gene set being downregulated in a specific tissue. The number in the squares shows the

normalized enrichment score. A and D) Enrichments in the different ALL and AML

subtypes with genes contained within gene sets from normal flow sorted hematopoietic

cells. Also included are gene sets from previously generated gene expression data of

pediatric ALL. B and E) Enrichments between gene sets derived from normal tissues and

genes downregulated in the different ALL and AML subtypes. C and F. Enrichments

between gene sets derived from different human cancers and genes downregulated in the

different ALL and AML subtypes.
