# Supplementary material for: Gene expression signatures in childhood acute leukemias are largely unique and distinct from those of normal tissues and other malignancies
Source: BMC Med Genomics. 2010 Mar 8;3:6. doi: 10.1186/1755-8794-3-6 (PMC2845086; doi:10.1186/1755-8794-3-6)
Supplement: Additional file 3 — GSEA reveals similarities between genes upregulated in pediatric APL with PML/RARA and those upregulated in normal promyelocytes. Heat maps and enrichment plots of the comparison of normal promyelocytes and APL. [file 1755-8794-3-6-S3.DOC]

**Additional file 3.**


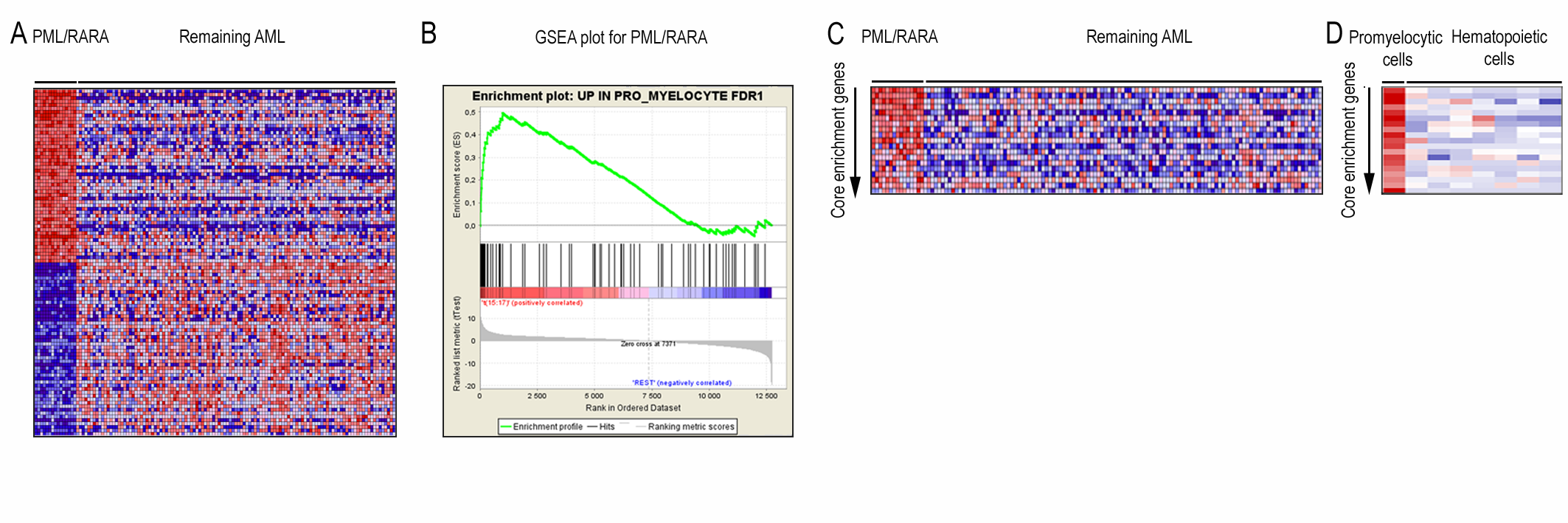


**Legend to additional file 3**. GSEA reveals similarities between genes upregulated in pediatric APL with *PML/RARA* and those upregulated in normal promyelocytes. A) Heat map of the top 50 up- and downregulated genes from the ranked gene list generated in GSEA on the pediatric AML data set. B) Enrichment plot showing enrichment of genes upregulated in promyelocytes among the top ranked upregulated genes in cases with PML/RARA. C) Heat map of the core enrichment genes in the AML data set. D) Heat map of the same core enrichment genes in the normal flow sorted hematopoietic cells. From figure C and D, the gene expression similarities of the *PML/RARA* and the normal promyelocytes can be easily appreciated.
