# Supplementary material for: Gene expression signatures in childhood acute leukemias are largely unique and distinct from those of normal tissues and other malignancies
Source: BMC Med Genomics. 2010 Mar 8;3:6. doi: 10.1186/1755-8794-3-6 (PMC2845086; doi:10.1186/1755-8794-3-6)
Supplement: Additional file 4 — GSEA reveals similarities between genes being upregulated in pediatric AML with AML M7 and those upregulated in normal MEP cells. Heat maps and enrichment plots of the comparison of normal MEP cells and AML M7. [file 1755-8794-3-6-S4.DOC]

**Additional file 4**


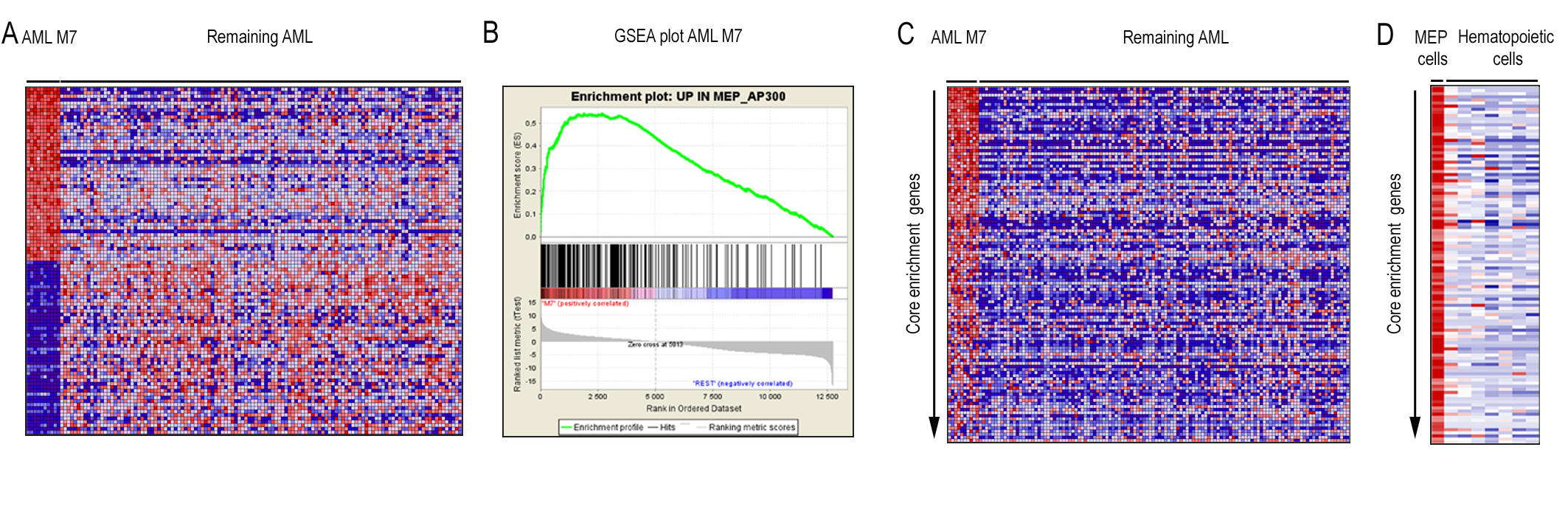


**Legend to additional file 4**. GSEA reveals similarities between genes being upregulated in pediatric AML with AML M7 and those upregulated in normal MEP cells. A) Heat map of the top 50 up- and downregulated genes from the ranked gene list generated in GSEA on the pediatric AML data set. B) Enrichment plot showing the enrichment of genes upregulated in MEP cells among the top ranked upregulated genes in cases with AML M7. C) Heat map of the core enrichment genes in the AML data set. D) Heat map of the same core enrichment genes in the normal flow sorted hematopoietic cells. From figure C and D, the similarities of the AML M7 and the normal MEP cells, can be easily appreciated.
