# Supplementary material for: Gene expression signatures in childhood acute leukemias are largely unique and distinct from those of normal tissues and other malignancies
Source: BMC Med Genomics. 2010 Mar 8;3:6. doi: 10.1186/1755-8794-3-6 (PMC2845086; doi:10.1186/1755-8794-3-6)
Supplement: Additional file 5 — Core enrichment genes in pediatric AML M7 when compared to genes being upregulated in normal flow sorted MEP cells. Table of the core enrichment genes, their rank and statistics from the gene set enrichment analysis. [file 1755-8794-3-6-S5.DOC]

**Additional file 5**. Core enrichment genes in pediatric AML M7 when compared to genes being upregulated in normal flow sorted MEP cells.

| *GENE SYMBOL* | *GENE TITLE* | *RANK IN GENE LIST* | *RANK METRIC SCORE* | *RUNNING ES* | *CORE ENRICHMENT* |
| --- | --- | --- | --- | --- | --- |
| ITGA2B | integrin, alpha 2b (platelet glycoprotein IIb of IIb/IIIa complex, antigen CD41) | 0 | 14.51153 | 0.023188 | Yes |
| TAL1 | T-cell acute lymphocytic leukemia 1 | 3 | 13.36094 | 0.044377 | Yes |
| HIGD1A | HIG1 domain family, member 1A | 4 | 12.85217 | 0.064914 | Yes |
| DLC1 | deleted in liver cancer 1 | 7 | 12.09897 | 0.084087 | Yes |
| MYL4 | myosin, light chain 4, alkali; atrial, embryonic | 18 | 9.555044 | 0.098555 | Yes |
| CMAS | cytidine monophosphate N-acetylneuraminic acid synthetase | 28 | 8.973001 | 0.112173 | Yes |
| TIMP3 | TIMP metallopeptidase inhibitor 3 (Sorsby fundus dystrophy, pseudoinflammatory) | 41 | 8.424876 | 0.124675 | Yes |
| PRUNE | prune homolog (Drosophila) | 58 | 7.770317 | 0.135811 | Yes |
| ATPIF1 | ATPase inhibitory factor 1 | 61 | 7.656745 | 0.147886 | Yes |
| DAG1 | dystroglycan 1 (dystrophin-associated glycoprotein 1) | 64 | 7.499591 | 0.159709 | Yes |
| TNIK | TRAF2 and NCK interacting kinase | 71 | 7.213398 | 0.170756 | Yes |
| ANK1 | ankyrin 1, erythrocytic | 86 | 6.897825 | 0.180658 | Yes |
| FKBP1B | FK506 binding protein 1B, 12.6 kDa | 99 | 6.761188 | 0.190501 | Yes |
| UROD | uroporphyrinogen decarboxylase | 106 | 6.651936 | 0.200651 | Yes |
| MYH10 | myosin, heavy chain 10, non-muscle | 111 | 6.600785 | 0.210878 | Yes |
| AGPAT1 | 1-acylglycerol-3-phosphate O-acyltransferase 1 (lysophosphatidic acid acyltransferase, alpha) | 124 | 6.411997 | 0.220164 | Yes |
| RCL1 | RNA terminal phosphate cyclase-like 1 | 132 | 6.324115 | 0.229709 | Yes |
| GFI1B | growth factor independent 1B (potential regulator of CDKN1A, translocated in CML) | 138 | 6.252155 | 0.239299 | Yes |
| RHAG | Rh-associated glycoprotein | 143 | 6.156332 | 0.248817 | Yes |
| FBXO7 | F-box protein 7 | 149 | 6.122262 | 0.258199 | Yes |
| GATA1 | GATA binding protein 1 (globin transcription factor 1) | 168 | 5.936654 | 0.266246 | Yes |
| SLC25A15 | solute carrier family 25 (mitochondrial carrier; ornithine transporter) member 15 | 169 | 5.907241 | 0.275685 | Yes |
| XK | X-linked Kx blood group (McLeod syndrome) | 182 | 5.794384 | 0.283984 | Yes |
| PRKAB1 | protein kinase, AMP-activated, beta 1 non-catalytic subunit | 191 | 5.669128 | 0.292402 | Yes |
| FHL2 | four and a half LIM domains 2 | 216 | 5.483419 | 0.299244 | Yes |
| PPOX | protoporphyrinogen oxidase | 224 | 5.381081 | 0.307283 | Yes |
| TPM1 | tropomyosin 1 (alpha) | 284 | 5.038279 | 0.310614 | Yes |
| CTNND1 | catenin (cadherin-associated protein), delta 1 | 285 | 5.018883 | 0.318634 | Yes |
| DNAJC6 | DnaJ (Hsp40) homolog, subfamily C, member 6 | 288 | 5.002996 | 0.326468 | Yes |
| RYR3 | ryanodine receptor 3 | 290 | 4.99428 | 0.334368 | Yes |
| ABCC4 | ATP-binding cassette, sub-family C (CFTR/MRP), member 4 | 292 | 4.984096 | 0.342252 | Yes |
| NAG | - | 296 | 4.971952 | 0.349957 | Yes |
| ROCK2 | Rho-associated, coiled-coil containing protein kinase 2 | 308 | 4.912176 | 0.356926 | Yes |
| UBAC1 | Null | 318 | 4.878294 | 0.364001 | Yes |
| CDC42BPA | CDC42 binding protein kinase alpha (DMPK-like) | 339 | 4.768039 | 0.37002 | Yes |
| BCCIP | BRCA2 and CDKN1A interacting protein | 363 | 4.678184 | 0.375656 | Yes |
| ARMC8 | armadillo repeat containing 8 | 380 | 4.599069 | 0.381724 | Yes |
| ORC4L | origin recognition complex, subunit 4-like (yeast) | 389 | 4.56809 | 0.388384 | Yes |
| HDHD3 | haloacid dehalogenase-like hydrolase domain containing 3 | 446 | 4.350657 | 0.390856 | Yes |
| SSBP3 | single stranded DNA binding protein 3 | 498 | 4.132286 | 0.393379 | Yes |
| SLC11A2 | solute carrier family 11 (proton-coupled divalent metal ion transporters), member 2 | 547 | 4.005822 | 0.395941 | Yes |
| VANGL1 | vang-like 1 (van gogh, Drosophila) | 566 | 3.966864 | 0.400839 | Yes |
| APBA2 | amyloid beta (A4) precursor protein-binding, family A, member 2 (X11-like) | 616 | 3.83944 | 0.403055 | Yes |
| KCNH2 | potassium voltage-gated channel, subfamily H (eag-related), member 2 | 625 | 3.812671 | 0.408507 | Yes |
| TFR2 | transferrin receptor 2 | 654 | 3.759214 | 0.412274 | Yes |
| GAS2L1 | growth arrest-specific 2 like 1 | 656 | 3.757792 | 0.418199 | Yes |
| SSB | Sjogren syndrome antigen B (autoantigen La) | 712 | 3.605967 | 0.419561 | Yes |
| PSEN2 | presenilin 2 (Alzheimer disease 4) | 714 | 3.601672 | 0.425236 | Yes |
| MRPS12 | mitochondrial ribosomal protein S12 | 721 | 3.590031 | 0.430492 | Yes |
| APEX2 | APEX nuclease (apurinic/apyrimidinic endonuclease) 2 | 779 | 3.493068 | 0.431514 | Yes |
| PNMT | phenylethanolamine N-methyltransferase | 783 | 3.483223 | 0.43684 | Yes |
| NR2F6 | nuclear receptor subfamily 2, group F, member 6 | 798 | 3.462982 | 0.441254 | Yes |
| NEDD4L | neural precursor cell expressed, developmentally down-regulated 4-like | 801 | 3.455568 | 0.446615 | Yes |
| STK25 | serine/threonine kinase 25 (STE20 homolog, yeast) | 825 | 3.407714 | 0.450221 | Yes |
| NARF | nuclear prelamin A recognition factor | 837 | 3.383476 | 0.454747 | Yes |
| CTNNA1 | catenin (cadherin-associated protein), alpha 1, 102kDa | 859 | 3.346852 | 0.458415 | Yes |
| CXADR | coxsackie virus and adenovirus receptor | 880 | 3.315495 | 0.462113 | Yes |
| NCKAP1 | NCK-associated protein 1 | 903 | 3.278836 | 0.465593 | Yes |
| TMOD1 | tropomodulin 1 | 913 | 3.263047 | 0.470087 | Yes |
| KEL | Kell blood group, metallo-endopeptidase | 916 | 3.261934 | 0.475139 | Yes |
| FLJ20489 | - | 937 | 3.219865 | 0.478684 | Yes |
| APOE | apolipoprotein E | 942 | 3.212323 | 0.483497 | Yes |
| CRYM | crystallin, mu | 972 | 3.146094 | 0.486204 | Yes |
| RNF123 | ring finger protein 123 | 999 | 3.106459 | 0.489088 | Yes |
| NSBP1 | nucleosomal binding protein 1 | 1014 | 3.086616 | 0.492901 | Yes |
| AMMECR1 | Alport syndrome, mental retardation, midface hypoplasia and elliptocytosis chromosomal region, gene 1 | 1050 | 3.026587 | 0.494937 | Yes |
| TSPAN6 | tetraspanin 6 | 1051 | 3.024605 | 0.49977 | Yes |
| ATP7B | ATPase, Cu++ transporting, beta polypeptide | 1063 | 3.009549 | 0.503699 | Yes |
| ELOVL6 | ELOVL family member 6, elongation of long chain fatty acids (FEN1/Elo2, SUR4/Elo3-like, yeast) | 1153 | 2.892194 | 0.501201 | Yes |
| WDR59 | WD repeat domain 59 | 1183 | 2.857995 | 0.503448 | Yes |
| SELENBP1 | selenium binding protein 1 | 1196 | 2.848192 | 0.507039 | Yes |
| GOSR2 | golgi SNAP receptor complex member 2 | 1205 | 2.840056 | 0.510937 | Yes |
| ALAD | aminolevulinate, delta-, dehydratase | 1218 | 2.827032 | 0.514495 | Yes |
| LEPR | leptin receptor | 1235 | 2.804291 | 0.517696 | Yes |
| LDB1 | LIM domain binding 1 | 1246 | 2.790777 | 0.521355 | Yes |
| MRS2L | MRS2-like, magnesium homeostasis factor (S. cerevisiae) | 1296 | 2.748824 | 0.521828 | Yes |
| RNASE1 | ribonuclease, RNase A family, 1 (pancreatic) | 1312 | 2.73332 | 0.524995 | Yes |
| TFRC | transferrin receptor (p90, CD71) | 1394 | 2.630579 | 0.522719 | Yes |
| MAP7 | microtubule-associated protein 7 | 1444 | 2.584692 | 0.52293 | Yes |
| ZFYVE21 | zinc finger, FYVE domain containing 21 | 1459 | 2.565148 | 0.525909 | Yes |
| SSX2IP | synovial sarcoma, X breakpoint 2 interacting protein | 1494 | 2.535368 | 0.52724 | Yes |
| NUP62CL | nucleoporin 62kDa C-terminal like | 1553 | 2.475064 | 0.526555 | Yes |
| ADAMTS3 | ADAM metallopeptidase with thrombospondin type 1 motif, 3 | 1557 | 2.471087 | 0.530264 | Yes |
| STXBP1 | syntaxin binding protein 1 | 1559 | 2.46753 | 0.534127 | Yes |
| PIR | pirin (iron-binding nuclear protein) | 1581 | 2.442957 | 0.536351 | Yes |
| ABCB6 | ATP-binding cassette, sub-family B (MDR/TAP), member 6 | 1620 | 2.409191 | 0.53716 | Yes |
| APEH | N-acylaminoacyl-peptide hydrolase | 1652 | 2.374111 | 0.538474 | Yes |
| HBS1L | HBS1-like (S. cerevisiae) | 1722 | 2.29328 | 0.536619 | Yes |
| GAD1 | glutamate decarboxylase 1 (brain, 67kDa) | 1823 | 2.205933 | 0.532145 | Yes |
| ABCC5 | ATP-binding cassette, sub-family C (CFTR/MRP), member 5 | 1852 | 2.172867 | 0.533377 | Yes |
| ADORA2B | adenosine A2b receptor | 1853 | 2.171895 | 0.536847 | Yes |
| ADD2 | adducin 2 (beta) | 1879 | 2.142269 | 0.53827 | Yes |
| TNFRSF25 | tumor necrosis factor receptor superfamily, member 25 | 1893 | 2.134929 | 0.540642 | Yes |
| TGM2 | transglutaminase 2 (C polypeptide, protein-glutamine-gamma-glutamyltransferase) | 1995 | 2.040499 | 0.535823 | Yes |
| GART | phosphoribosylglycinamide formyltransferase, phosphoribosylglycinamide synthetase, phosphoribosylaminoimidazole synthetase | 2029 | 2.019078 | 0.53641 | Yes |
| MED6 | mediator of RNA polymerase II transcription, subunit 6 homolog (S. cerevisiae) | 2077 | 1.971703 | 0.5358 | Yes |
| UNC13B | unc-13 homolog B (C. elegans) | 2091 | 1.961279 | 0.537894 | Yes |
| NUP98 | nucleoporin 98kDa | 2118 | 1.942859 | 0.538919 | Yes |
| AKR1C2 | aldo-keto reductase family 1, member C2 (dihydrodiol dehydrogenase 2; bile acid binding protein; 3-alpha hydroxysteroid dehydrogenase, type III) | 2173 | 1.899244 | 0.537634 | Yes |
| MUC1 | mucin 1, cell surface associated | 2244 | 1.842262 | 0.534978 | Yes |
| FUT1 | fucosyltransferase 1 (galactoside 2-alpha-L-fucosyltransferase, H blood group) | 2249 | 1.838761 | 0.537597 | Yes |
| EPOR | erythropoietin receptor | 2329 | 1.783935 | 0.534128 | Yes |
| PKLR | pyruvate kinase, liver and RBC | 2350 | 1.768587 | 0.535354 | Yes |
| PTGES2 | prostaglandin E synthase 2 | 2424 | 1.721279 | 0.532265 | Yes |
| RAP2B | RAP2B, member of RAS oncogene family | 2472 | 1.686177 | 0.531199 | Yes |
| PPT2 | palmitoyl-protein thioesterase 2 | 2488 | 1.67498 | 0.532676 | Yes |
| PTGER3 | prostaglandin E receptor 3 (subtype EP3) | 2506 | 1.657244 | 0.533964 | Yes |
| BCKDHB | branched chain keto acid dehydrogenase E1, beta polypeptide (maple syrup urine disease) | 2509 | 1.65438 | 0.536448 | Yes |
| FGFR3 | fibroblast growth factor receptor 3 (achondroplasia, thanatophoric dwarfism) | 2519 | 1.651792 | 0.538367 | Yes |
| SPTBN2 | spectrin, beta, non-erythrocytic 2 | 2569 | 1.601146 | 0.537006 | Yes |
| NLE1 | notchless homolog 1 (Drosophila) | 2605 | 1.576824 | 0.536726 | Yes |
| RIF1 | RAP1 interacting factor homolog (yeast) | 2649 | 1.548836 | 0.535761 | Yes |
| NMU | neuromedin U | 2650 | 1.547985 | 0.538234 | Yes |
| PCTK1 | PCTAIRE protein kinase 1 | 2663 | 1.540314 | 0.539736 | Yes |
| GBAS | glioblastoma amplified sequence | 2668 | 1.536994 | 0.541872 | Yes |
