# Supplementary material for: Gene expression signatures in childhood acute leukemias are largely unique and distinct from those of normal tissues and other malignancies
Source: BMC Med Genomics. 2010 Mar 8;3:6. doi: 10.1186/1755-8794-3-6 (PMC2845086; doi:10.1186/1755-8794-3-6)
Supplement: Additional file 6 — Core enrichment genes in pediatric ALL with 11q23/MLL when compared to genes being upregulated in normal bronchoepitelial cells. Table of the core enrichment genes, their rank and statistics from the gene set enrichment analysis. [file 1755-8794-3-6-S6.DOC]

**Additional file 6**. Core enrichment genes in pediatric ALL with 11q23/*MLL* when compared to genes being upregulated in normal bronchoepitelial cells.

| *GENE SYMBOL* | *GENE TITLE* | *RANK IN GENE LIST* | *RANK METRIC SCORE* | *RUNNING ES* | *CORE ENRICHMENT* |
| --- | --- | --- | --- | --- | --- |
| RRAS2 | related RAS viral (r-ras) oncogene homolog 2 | 75 | 7.213545 | 0.007592 | Yes |
| ADK | adenosine kinase | 96 | 6.80792 | 0.017613 | Yes |
| AK2 | adenylate kinase 2 | 208 | 5.585423 | 0.020514 | Yes |
| RBM13 | RNA binding motif protein 13 | 281 | 5.158715 | 0.02491 | Yes |
| MINA | MYC induced nuclear antigen | 419 | 4.728477 | 0.024945 | Yes |
| KIAA0020 | KIAA0020 | 443 | 4.636464 | 0.031242 | Yes |
| DPP3 | dipeptidyl-peptidase 3 | 527 | 4.417155 | 0.033805 | Yes |
| FAM82B | family with sequence similarity 82, member B | 534 | 4.402534 | 0.040676 | Yes |
| CDK7 | cyclin-dependent kinase 7 (MO15 homolog, Xenopus laevis, cdk-activating kinase) | 555 | 4.322985 | 0.046629 | Yes |
| DIMT1L | DIM1 dimethyladenosine transferase 1-like (S. cerevisiae) | 572 | 4.287913 | 0.052749 | Yes |
| KCTD5 | potassium channel tetramerisation domain containing 5 | 605 | 4.217035 | 0.057853 | Yes |
| STAMBP | STAM binding protein | 656 | 4.09672 | 0.061748 | Yes |
| GTPBP4 | GTP binding protein 4 | 657 | 4.095953 | 0.068454 | Yes |
| XTP3TPA | - | 678 | 4.063338 | 0.073982 | Yes |
| HMOX2 | heme oxygenase (decycling) 2 | 689 | 4.019531 | 0.080001 | Yes |
| PDCD5 | programmed cell death 5 | 697 | 4.005077 | 0.086164 | Yes |
| DNAJA2 | DnaJ (Hsp40) homolog, subfamily A, member 2 | 727 | 3.954952 | 0.091008 | Yes |
| SRP72 | signal recognition particle 72kDa | 777 | 3.865252 | 0.09458 | Yes |
| UCHL3 | ubiquitin carboxyl-terminal esterase L3 (ubiquitin thiolesterase) | 792 | 3.842054 | 0.100083 | Yes |
| ETF1 | eukaryotic translation termination factor 1 | 798 | 3.829784 | 0.106072 | Yes |
| PXMP3 | peroxisomal membrane protein 3, 35kDa (Zellweger syndrome) | 813 | 3.807785 | 0.111519 | Yes |
| RTCD1 | RNA terminal phosphate cyclase domain 1 | 824 | 3.792958 | 0.117167 | Yes |
| MAN2A1 | mannosidase, alpha, class 2A, member 1 | 846 | 3.748506 | 0.122123 | Yes |
| MTMR2 | myotubularin related protein 2 | 859 | 3.725828 | 0.127548 | Yes |
| HMGA2 | high mobility group AT-hook 2 | 894 | 3.676498 | 0.131654 | Yes |
| ZNF593 | zinc finger protein 593 | 907 | 3.657822 | 0.136968 | Yes |
| APTX | aprataxin | 923 | 3.635432 | 0.142077 | Yes |
| EI24 | etoposide induced 2.4 mRNA | 953 | 3.576752 | 0.146301 | Yes |
| PNO1 | null | 977 | 3.545007 | 0.150812 | Yes |
| POLR2G | polymerase (RNA) II (DNA directed) polypeptide G | 1032 | 3.477928 | 0.153468 | Yes |
| RAP2B | RAP2B, member of RAS oncogene family | 1037 | 3.473375 | 0.15893 | Yes |
| NARG1 | NMDA receptor regulated 1 | 1048 | 3.460894 | 0.164034 | Yes |
| ITPA | inosine triphosphatase (nucleoside triphosphate pyrophosphatase) | 1074 | 3.423992 | 0.168234 | Yes |
| WDR41 | WD repeat domain 41 | 1080 | 3.416601 | 0.173546 | Yes |
| FDFT1 | farnesyl-diphosphate farnesyltransferase 1 | 1118 | 3.361718 | 0.176969 | Yes |
| EBNA1BP2 | EBNA1 binding protein 2 | 1276 | 3.154841 | 0.173303 | Yes |
| TXNDC9 | thioredoxin domain containing 9 | 1355 | 3.072037 | 0.173945 | Yes |
| PPA2 | pyrophosphatase (inorganic) 2 | 1453 | 2.970559 | 0.173352 | Yes |
| RAP1GDS1 | RAP1, GTP-GDP dissociation stimulator 1 | 1494 | 2.926052 | 0.175892 | Yes |
| HBEGF | heparin-binding EGF-like growth factor | 1499 | 2.921418 | 0.180451 | Yes |
| GOLT1B | golgi transport 1 homolog B (S. cerevisiae) | 1505 | 2.918226 | 0.184947 | Yes |
| HMGCR | 3-hydroxy-3-methylglutaryl-Coenzyme A reductase | 1541 | 2.882119 | 0.187697 | Yes |
| POP4 | processing of precursor 4, ribonuclease P/MRP subunit (S. cerevisiae) | 1574 | 2.860013 | 0.19058 | Yes |
| METAP1 | methionyl aminopeptidase 1 | 1599 | 2.841769 | 0.193882 | Yes |
| YKT6 | YKT6 v-SNARE homolog (S. cerevisiae) | 1615 | 2.827955 | 0.197669 | Yes |
| DNAJC7 | DnaJ (Hsp40) homolog, subfamily C, member 7 | 1626 | 2.820371 | 0.201724 | Yes |
| AVEN | apoptosis, caspase activation inhibitor | 1655 | 2.799415 | 0.204732 | Yes |
| NUP37 | nucleoporin 37kDa | 1679 | 2.777468 | 0.207986 | Yes |
| GM2A | GM2 ganglioside activator | 1681 | 2.776322 | 0.212475 | Yes |
| KIAA0090 | KIAA0090 | 1696 | 2.759938 | 0.216206 | Yes |
| TCP1 | t-complex 1 | 1771 | 2.676754 | 0.216426 | Yes |
| CSTA | cystatin A (stefin A) | 1793 | 2.660812 | 0.219601 | Yes |
| DNM1L | dynamin 1-like | 1889 | 2.585489 | 0.21849 | Yes |
| TWF1 | null | 1942 | 2.547175 | 0.219736 | Yes |
| CEBPG | CCAAT/enhancer binding protein (C/EBP), gamma | 1944 | 2.546445 | 0.223849 | Yes |
| GCLC | glutamate-cysteine ligase, catalytic subunit | 2063 | 2.473052 | 0.22126 | Yes |
| SEC23B | Sec23 homolog B (S. cerevisiae) | 2089 | 2.454116 | 0.223872 | Yes |
| SAMM50 | sorting and assembly machinery component 50 homolog (S. cerevisiae) | 2099 | 2.444335 | 0.227367 | Yes |
| MAPBPIP | - | 2160 | 2.399885 | 0.227921 | Yes |
| ABCE1 | ATP-binding cassette, sub-family E (OABP), member 1 | 2254 | 2.330811 | 0.226506 | Yes |
| GRPEL1 | GrpE-like 1, mitochondrial (E. coli) | 2278 | 2.311815 | 0.228997 | Yes |
| AFG3L2 | AFG3 ATPase family gene 3-like 2 (yeast) | 2279 | 2.310178 | 0.23278 | Yes |
| TXNDC1 | thioredoxin domain containing 1 | 2297 | 2.300305 | 0.23559 | Yes |
| TGFA | transforming growth factor, alpha | 2299 | 2.299047 | 0.239298 | Yes |
| TRIAP1 | TP53 regulated inhibitor of apoptosis 1 | 2376 | 2.248111 | 0.238703 | Yes |
| EXT2 | exostoses (multiple) 2 | 2384 | 2.240239 | 0.241977 | Yes |
| MAK10 | MAK10 homolog, amino-acid N-acetyltransferase subunit, (S. cerevisiae) | 2456 | 2.193608 | 0.241575 | Yes |
| TIPARP | TCDD-inducible poly(ADP-ribose) polymerase | 2540 | 2.141758 | 0.240413 | Yes |
| XAB1 | XPA binding protein 1, GTPase | 2578 | 2.114691 | 0.241793 | Yes |
| SQLE | squalene epoxidase | 2592 | 2.106991 | 0.244512 | Yes |
| TSR1 | TSR1, 20S rRNA accumulation, homolog (S. cerevisiae) | 2599 | 2.101923 | 0.247616 | Yes |
| PGK1 | phosphoglycerate kinase 1 | 2629 | 2.082091 | 0.249393 | Yes |
| FTSJ1 | FtsJ homolog 1 (E. coli) | 2654 | 2.067773 | 0.251429 | Yes |
| ADRB2 | adrenergic, beta-2-, receptor, surface | 2656 | 2.067659 | 0.254758 | Yes |
| COG5 | component of oligomeric golgi complex 5 | 2686 | 2.051038 | 0.256485 | Yes |
| PA2G4 | proliferation-associated 2G4, 38kDa | 2714 | 2.027907 | 0.258286 | Yes |
| EIF2B4 | eukaryotic translation initiation factor 2B, subunit 4 delta, 67kDa | 2751 | 2.009578 | 0.259551 | Yes |
| PPIE | peptidylprolyl isomerase E (cyclophilin E) | 2786 | 1.992096 | 0.2609 | Yes |
| UTX | ubiquitously transcribed tetratricopeptide repeat, X chromosome | 2799 | 1.980873 | 0.263468 | Yes |
| SYNCRIP | synaptotagmin binding, cytoplasmic RNA interacting protein | 2802 | 1.97801 | 0.266594 | Yes |
| LIMA1 | LIM domain and actin binding 1 | 2811 | 1.972575 | 0.269374 | Yes |
| KYNU | kynureninase (L-kynurenine hydrolase) | 2828 | 1.965096 | 0.271691 | Yes |
| DSE | dermatan sulfate epimerase | 2840 | 1.960686 | 0.274283 | Yes |
| LSM3 | LSM3 homolog, U6 small nuclear RNA associated (S. cerevisiae) | 2841 | 1.960433 | 0.277492 | Yes |
| UMPS | uridine monophosphate synthetase (orotate phosphoribosyl transferase and orotidine-5'-decarboxylase) | 2856 | 1.952456 | 0.279902 | Yes |
| CLP1 | CLP1, cleavage and polyadenylation factor I subunit, homolog (S. cerevisiae) | 2889 | 1.928421 | 0.281259 | Yes |
| CSNK2A1 | casein kinase 2, alpha 1 polypeptide | 2919 | 1.906001 | 0.282748 | Yes |
| HRAS | v-Ha-ras Harvey rat sarcoma viral oncogene homolog | 2995 | 1.864717 | 0.281582 | Yes |
| ERGIC2 | ERGIC and golgi 2 | 3021 | 1.852354 | 0.283209 | Yes |
| PSME3 | proteasome (prosome, macropain) activator subunit 3 (PA28 gamma; Ki) | 3137 | 1.7781 | 0.279651 | Yes |
| ETHE1 | ethylmalonic encephalopathy 1 | 3289 | 1.701103 | 0.273942 | Yes |
| NDUFS1 | NADH dehydrogenase (ubiquinone) Fe-S protein 1, 75kDa (NADH-coenzyme Q reductase) | 3293 | 1.697705 | 0.276553 | Yes |
| TIMM17A | translocase of inner mitochondrial membrane 17 homolog A (yeast) | 3307 | 1.690527 | 0.278589 | Yes |
| SSH3 | slingshot homolog 3 (Drosophila) | 3400 | 1.645917 | 0.276109 | Yes |
| EMG1 | EMG1 nucleolar protein homolog (S. cerevisiae) | 3402 | 1.64354 | 0.278744 | Yes |
| ZNHIT3 | zinc finger, HIT type 3 | 3418 | 1.638566 | 0.280583 | Yes |
| SARS | seryl-tRNA synthetase | 3435 | 1.632259 | 0.282355 | Yes |
| MRPL13 | mitochondrial ribosomal protein L13 | 3443 | 1.625247 | 0.284622 | Yes |
| CLTB | clathrin, light chain (Lcb) | 3494 | 1.592724 | 0.284417 | Yes |
| KLF7 | Kruppel-like factor 7 (ubiquitous) | 3500 | 1.590406 | 0.28674 | Yes |
| SLC25A24 | solute carrier family 25 (mitochondrial carrier; phosphate carrier), member 24 | 3536 | 1.575013 | 0.28735 | Yes |
| ELOVL1 | elongation of very long chain fatty acids (FEN1/Elo2, SUR4/Elo3, yeast)-like 1 | 3610 | 1.536058 | 0.285758 | Yes |
| LRRC40 | leucine rich repeat containing 40 | 3667 | 1.503235 | 0.285069 | Yes |
| NEDD8 | neural precursor cell expressed, developmentally down-regulated 8 | 3704 | 1.484029 | 0.285474 | Yes |
| GEMIN6 | gem (nuclear organelle) associated protein 6 | 3729 | 1.469989 | 0.286531 | Yes |
| CNIH4 | cornichon homolog 4 (Drosophila) | 3734 | 1.466473 | 0.288707 | Yes |
| PTRH2 | peptidyl-tRNA hydrolase 2 | 3775 | 1.445204 | 0.288823 | Yes |
| ANXA2P3 | annexin A2 pseudogene 3 | 3786 | 1.442041 | 0.290621 | Yes |
