# Supplementary material for: Gene expression signatures in childhood acute leukemias are largely unique and distinct from those of normal tissues and other malignancies
Source: BMC Med Genomics. 2010 Mar 8;3:6. doi: 10.1186/1755-8794-3-6 (PMC2845086; doi:10.1186/1755-8794-3-6)
Supplement: Additional file 7 — Core enrichment genes in pediatric ALL with 11q23/MLL when compared to genes being upregulated in normal adipocytes. Table of the core enrichment genes, their rank and statistics from the gene set enrichment analysis. [file 1755-8794-3-6-S7.DOC]

**Additional file 7**. Core enrichment genes in pediatric ALL with 11q23/*MLL* when compared to genes being upregulated in normal adipocytes.

| *GENE SYMBOL* | *GENE TITLE* | *RANK IN GENE LIST* | *RANK METRIC SCORE* | *RUNNING ES* | *CORE ENRICHMENT* |
| --- | --- | --- | --- | --- | --- |
| ARL6IP5 | ADP-ribosylation-like factor 6 interacting protein 5 | 11 | 10.58212 | 0.016478 | Yes |
| MPZL1 | myelin protein zero-like 1 | 20 | 9.757732 | 0.031792 | Yes |
| HK2 | hexokinase 2 | 44 | 8.205438 | 0.043755 | Yes |
| SCP2 | sterol carrier protein 2 | 84 | 7.067068 | 0.052979 | Yes |
| DIAPH2 | diaphanous homolog 2 (Drosophila) | 93 | 6.853565 | 0.063602 | Yes |
| ACSL1 | acyl-CoA synthetase long-chain family member 1 | 104 | 6.657106 | 0.073794 | Yes |
| HADH | hydroxyacyl-Coenzyme A dehydrogenase | 108 | 6.608123 | 0.084302 | Yes |
| PIGF | phosphatidylinositol glycan anchor biosynthesis, class F | 125 | 6.436738 | 0.093801 | Yes |
| S100A4 | S100 calcium binding protein A4 | 135 | 6.342331 | 0.103541 | Yes |
| CD302 | CD302 molecule | 150 | 6.141582 | 0.112676 | Yes |
| DBI | diazepam binding inhibitor (GABA receptor modulator, acyl-Coenzyme A binding protein) | 166 | 5.998223 | 0.121523 | Yes |
| ARPC4 | actin related protein 2/3 complex, subunit 4, 20kDa | 189 | 5.776502 | 0.129618 | Yes |
| RPS27L | ribosomal protein S27-like | 201 | 5.663649 | 0.13815 | Yes |
| DECR1 | 2,4-dienoyl CoA reductase 1, mitochondrial | 217 | 5.527411 | 0.146236 | Yes |
| GREM1 | gremlin 1, cysteine knot superfamily, homolog (Xenopus laevis) | 225 | 5.456976 | 0.154659 | Yes |
| WIPI1 | WD repeat domain, phosphoinositide interacting 1 | 275 | 5.224235 | 0.160343 | Yes |
| CEBPA | CCAAT/enhancer binding protein (C/EBP), alpha | 277 | 5.191732 | 0.168675 | Yes |
| GLUL | glutamate-ammonia ligase (glutamine synthetase) | 304 | 5.076821 | 0.175415 | Yes |
| RHOQ | ras homolog gene family, member Q | 305 | 5.075725 | 0.183615 | Yes |
| SNX3 | sorting nexin 3 | 306 | 5.074287 | 0.191813 | Yes |
| GLT25D1 | glycosyltransferase 25 domain containing 1 | 307 | 5.073689 | 0.20001 | Yes |
| CYB5R3 | cytochrome b5 reductase 3 | 417 | 4.730722 | 0.201523 | Yes |
| CS | citrate synthase | 427 | 4.675756 | 0.208571 | Yes |
| ABHD4 | abhydrolase domain containing 4 | 432 | 4.663318 | 0.21588 | Yes |
| TSPAN3 | tetraspanin 3 | 482 | 4.542618 | 0.220463 | Yes |
| MPV17 | MpV17 mitochondrial inner membrane protein | 516 | 4.440724 | 0.225782 | Yes |
| DIMT1L | DIM1 dimethyladenosine transferase 1-like (S. cerevisiae) | 572 | 4.287913 | 0.229616 | Yes |
| ACYP2 | acylphosphatase 2, muscle type | 573 | 4.287912 | 0.236544 | Yes |
| GBE1 | glucan (1,4-alpha-), branching enzyme 1 (glycogen branching enzyme, Andersen disease, glycogen storage disease type IV) | 612 | 4.198068 | 0.241189 | Yes |
| FKBP5 | FK506 binding protein 5 | 723 | 3.962393 | 0.241405 | Yes |
| PGD | phosphogluconate dehydrogenase | 855 | 3.732507 | 0.240069 | Yes |
| ECHDC1 | enoyl Coenzyme A hydratase domain containing 1 | 1036 | 3.475579 | 0.235562 | Yes |
| PALLD | palladin, cytoskeletal associated protein | 1114 | 3.364475 | 0.236667 | Yes |
| FASN | fatty acid synthase | 1136 | 3.341479 | 0.240885 | Yes |
| PTGS1 | prostaglandin-endoperoxide synthase 1 (prostaglandin G/H synthase and cyclooxygenase) | 1163 | 3.309717 | 0.24477 | Yes |
| MYCBP2 | MYC binding protein 2 | 1187 | 3.276244 | 0.248769 | Yes |
| LDHA | lactate dehydrogenase A | 1202 | 3.260224 | 0.253249 | Yes |
| RETSAT | retinol saturase (all-trans-retinol 13,14-reductase) | 1214 | 3.237509 | 0.257861 | Yes |
| LOC727942 | - | 1317 | 3.109995 | 0.25715 | Yes |
| ACLY | ATP citrate lyase | 1391 | 3.030346 | 0.25794 | Yes |
| GLIPR1 | GLI pathogenesis-related 1 (glioma) | 1518 | 2.903382 | 0.255545 | Yes |
| SORT1 | sortilin 1 | 1632 | 2.812414 | 0.253735 | Yes |
| HADHA | hydroxyacyl-Coenzyme A dehydrogenase/3-ketoacyl-Coenzyme A thiolase/enoyl-Coenzyme A hydratase (trifunctional protein), alpha subunit | 1705 | 2.753278 | 0.254134 | Yes |
| AACS | acetoacetyl-CoA synthetase | 1742 | 2.70201 | 0.256475 | Yes |
| VCAN | versican | 1743 | 2.701573 | 0.260839 | Yes |
| PNPLA2 | patatin-like phospholipase domain containing 2 | 1807 | 2.64643 | 0.261572 | Yes |
| ACACA | acetyl-Coenzyme A carboxylase alpha | 1816 | 2.63724 | 0.265383 | Yes |
| DEXI | - | 2007 | 2.511439 | 0.258756 | Yes |
| FOXO3 | forkhead box O3 | 2093 | 2.44856 | 0.257932 | Yes |
| MAP2K2 | mitogen-activated protein kinase kinase 2 | 2115 | 2.434552 | 0.260684 | Yes |
| PDGFD | platelet derived growth factor D | 2203 | 2.370659 | 0.259622 | Yes |
| STON1 | stonin 1 | 2207 | 2.368921 | 0.26328 | Yes |
| TBC1D16 | TBC1 domain family, member 16 | 2230 | 2.350186 | 0.26584 | Yes |
| OSBPL1A | oxysterol binding protein-like 1A | 2293 | 2.303232 | 0.266075 | Yes |
| SLC7A6 | solute carrier family 7 (cationic amino acid transporter, y+ system), member 6 | 2389 | 2.236005 | 0.264345 | Yes |
| CLIC4 | chloride intracellular channel 4 | 2403 | 2.225582 | 0.267209 | Yes |
| PTPN11 | protein tyrosine phosphatase, non-receptor type 11 (Noonan syndrome 1) | 2476 | 2.185959 | 0.266692 | Yes |
| CCDC69 | coiled-coil domain containing 69 | 2498 | 2.169171 | 0.269016 | Yes |
