# Supplementary material for: Gene expression signatures in childhood acute leukemias are largely unique and distinct from those of normal tissues and other malignancies
Source: BMC Med Genomics. 2010 Mar 8;3:6. doi: 10.1186/1755-8794-3-6 (PMC2845086; doi:10.1186/1755-8794-3-6)
Supplement: Additional file 8 — Core enrichment genes in pediatric ALL with MLL when compared to genes being upregulated in normal tissue derived from prostate. Table of the core enrichment genes, their rank and statistics from the gene set enrichment analysis. [file 1755-8794-3-6-S8.DOC]

**Additional file 8** Core enrichment genes in pediatric ALLs with *MLL* when compared to genes being upregulated in normal tissue derived from prostate.

| *GENE SYMBOL* | *GENETITLE* | *RANK IN GENE LIST* | *RANK METRIC SCORE* | *RUNNING ES* | *CORE ENRICHMENT* |
| --- | --- | --- | --- | --- | --- |
| IKBKB | inhibitor of kappa light polypeptide gene enhancer in B-cells, kinase beta | 43 | 8.241756 | 0.012221 | Yes |
| SIDT2 | SID1 transmembrane family, member 2 | 61 | 7.420974 | 0.024447 | Yes |
| METTL7A | methyltransferase like 7A | 80 | 7.167294 | 0.036166 | Yes |
| TMBIM4 | transmembrane BAX inhibitor motif containing 4 | 92 | 6.888665 | 0.047783 | Yes |
| ASMTL | acetylserotonin O-methyltransferase-like | 214 | 5.55079 | 0.050838 | Yes |
| DDT | D-dopachrome tautomerase | 273 | 5.228689 | 0.056864 | Yes |
| SORD | sorbitol dehydrogenase | 284 | 5.146807 | 0.065444 | Yes |
| MAP7 | microtubule-associated protein 7 | 290 | 5.124664 | 0.074266 | Yes |
| RER1 | RER1 retention in endoplasmic reticulum 1 homolog (S. cerevisiae) | 302 | 5.087649 | 0.082684 | Yes |
| DAP | death-associated protein | 314 | 5.04466 | 0.091026 | Yes |
| DIP | - | 322 | 5.014752 | 0.09954 | Yes |
| PIGH | phosphatidylinositol glycan anchor biosynthesis, class H | 337 | 4.958539 | 0.107561 | Yes |
| IGF2R | insulin-like growth factor 2 receptor | 398 | 4.781873 | 0.11268 | Yes |
| GUCY1A3 | guanylate cyclase 1, soluble, alpha 3 | 433 | 4.66281 | 0.119051 | Yes |
| GMDS | GDP-mannose 4,6-dehydratase | 464 | 4.575083 | 0.12549 | Yes |
| PAK1IP1 | PAK1 interacting protein 1 | 470 | 4.563056 | 0.133314 | Yes |
| MRP63 | mitochondrial ribosomal protein 63 | 480 | 4.548607 | 0.140888 | Yes |
| VPS13D | vacuolar protein sorting 13 homolog D (S. cerevisiae) | 500 | 4.482456 | 0.147781 | Yes |
| MPG | N-methylpurine-DNA glycosylase | 574 | 4.286725 | 0.15129 | Yes |
| PLEKHB2 | pleckstrin homology domain containing, family B (evectins) member 2 | 579 | 4.271941 | 0.158653 | Yes |
| FLJ22222 | - | 633 | 4.155565 | 0.163054 | Yes |
| CD320 | CD320 molecule | 663 | 4.08216 | 0.168675 | Yes |
| HMG20B | high-mobility group 20B | 665 | 4.080364 | 0.175866 | Yes |
| AKT1 | v-akt murine thymoma viral oncogene homolog 1 | 893 | 3.677704 | 0.169633 | Yes |
| RPL13 | ribosomal protein L13 | 917 | 3.638792 | 0.174803 | Yes |
| RPS15 | ribosomal protein S15 | 926 | 3.631266 | 0.180803 | Yes |
| SCAND1 | SCAN domain containing 1 | 946 | 3.589612 | 0.186111 | Yes |
| CTBS | chitobiase, di-N-acetyl- | 966 | 3.556182 | 0.191359 | Yes |
| DHRS7 | dehydrogenase/reductase (SDR family) member 7 | 978 | 3.54498 | 0.197037 | Yes |
| ABHD11 | abhydrolase domain containing 11 | 1019 | 3.491146 | 0.200989 | Yes |
| EID1 | EP300 interacting inhibitor of differentiation 1 | 1095 | 3.390264 | 0.202793 | Yes |
| RABEP2 | rabaptin, RAB GTPase binding effector protein 2 | 1176 | 3.287483 | 0.204134 | Yes |
| SFRS11 | splicing factor, arginine/serine-rich 11 | 1203 | 3.258836 | 0.20846 | Yes |
| PPCS | phosphopantothenoylcysteine synthetase | 1204 | 3.257501 | 0.214247 | Yes |
| PPAP2A | phosphatidic acid phosphatase type 2A | 1321 | 3.107201 | 0.213242 | Yes |
| BRP44 | brain protein 44 | 1517 | 2.904123 | 0.207435 | Yes |
| OCEL1 | occludin/ELL domain containing 1 | 1616 | 2.825634 | 0.206943 | Yes |
| PDPK1 | 3-phosphoinositide dependent protein kinase-1 | 1623 | 2.822948 | 0.21162 | Yes |
| SC5DL | sterol-C5-desaturase (ERG3 delta-5-desaturase homolog, fungal)-like | 1640 | 2.806852 | 0.215705 | Yes |
| SOCS2 | suppressor of cytokine signaling 2 | 1643 | 2.805077 | 0.220576 | Yes |
| KIAA0152 | KIAA0152 | 1668 | 2.785904 | 0.224174 | Yes |
| CSNK1D | casein kinase 1, delta | 1685 | 2.770905 | 0.228197 | Yes |
| ZZEF1 | zinc finger, ZZ-type with EF-hand domain 1 | 1845 | 2.618075 | 0.223905 | Yes |
| VPS37C | vacuolar protein sorting 37 homolog C (S. cerevisiae) | 1931 | 2.555077 | 0.223664 | Yes |
| PPP3CA | protein phosphatase 3 (formerly 2B), catalytic subunit, alpha isoform (calcineurin A alpha) | 1939 | 2.549433 | 0.227799 | Yes |
| SAFB2 | scaffold attachment factor B2 | 1941 | 2.547962 | 0.232268 | Yes |
| LRRFIP1 | leucine rich repeat (in FLII) interacting protein 1 | 1955 | 2.539799 | 0.236048 | Yes |
| RAB4A | RAB4A, member RAS oncogene family | 1994 | 2.522475 | 0.238392 | Yes |
| CCDC47 | coiled-coil domain containing 47 | 2045 | 2.485283 | 0.239995 | Yes |
| SLC35A3 | solute carrier family 35 (UDP-N-acetylglucosamine (UDP-GlcNAc) transporter), member A3 | 2111 | 2.436031 | 0.240666 | Yes |
| USP20 | ubiquitin specific peptidase 20 | 2119 | 2.43213 | 0.244593 | Yes |
| TCF25 | transcription factor 25 (basic helix-loop-helix) | 2257 | 2.327413 | 0.241023 | Yes |
| HERPUD1 | homocysteine-inducible, endoplasmic reticulum stress-inducible, ubiquitin-like domain member 1 | 2307 | 2.295925 | 0.242345 | Yes |
| AAK1 | AP2 associated kinase 1 | 2341 | 2.271391 | 0.244524 | Yes |
| DEGS1 | degenerative spermatocyte homolog 1, lipid desaturase (Drosophila) | 2414 | 2.219746 | 0.244418 | Yes |
| RALB | v-ral simian leukemia viral oncogene homolog B (ras related; GTP binding protein) | 2439 | 2.201499 | 0.246979 | Yes |
