# Supplementary material for: Gene expression signatures in childhood acute leukemias are largely unique and distinct from those of normal tissues and other malignancies
Source: BMC Med Genomics. 2010 Mar 8;3:6. doi: 10.1186/1755-8794-3-6 (PMC2845086; doi:10.1186/1755-8794-3-6)
Supplement: Additional file 9 — Isolation strategy of the different subpopulations analyzed for gene expression. Example of the isolation strategy of the hematopoietic subpopulations. [file 1755-8794-3-6-S9.DOC]

**Additional file 9**


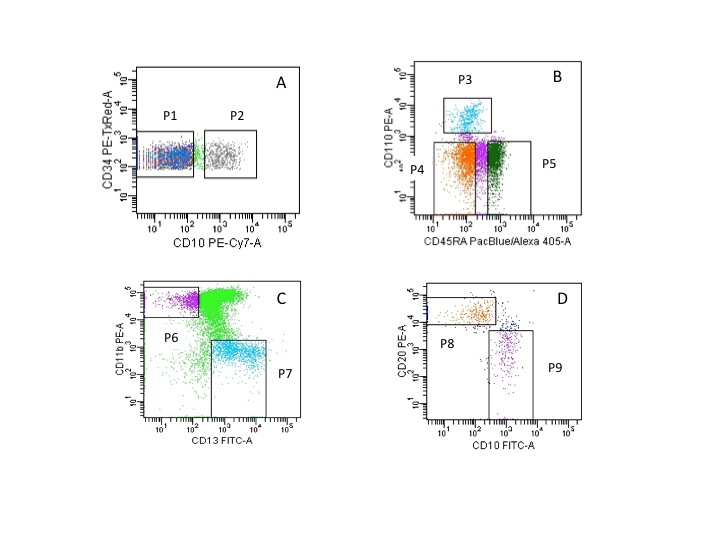


**Legend to additional file 9.** Example of isolation of the different subpopulations analyzed for gene expression. Panel A shows CD34^+^/CD19^-^/CD10^-^ in P1 and CD34^+^/CD19^+^/CD10^+^ cells in P2 (pro-B-cells). Panel B shows the further separation of cells from P1 into CD34^+^/CD123^low^/CD45RA^-^/CD110^+^ (P3, megakaryocyte/erythrocyte progenitors, MEP), CD34^+^/CD123^low^/CD45RA^-^/CD110^-^ (P4, common myeloid progenitors, CMP), and CD34^+^/CD123^low^/CD45RA^+^/CD110^-^ (P5, granulocyte/macrophage progenitors, GMP). Panel C shows isolation of two different myeloid maturation stages in CD34^-^/CD117^-^/CD33^+^/CD13^low^/CD11b^++^/CD14^-^/CD2^-^ (P6, corresponding to myelocytes and metamyelocytes), and CD34^-^/CD117^low^/CD33^+^/CD13^+^/CD11b^low^/CD2^-^ (P7, enriched for promyelocytes). Panel D shows the isolation of two B-lymphocyte differentiation stages in CD34^-^/CD19^+^/CD10^low^/CD20^++^ (P8, immature B-cells), and in CD34^-^/CD19^+^/CD10^+^/CD20^low^ (P9, pre-B-cells).
