# Supplementary material for: Gene expression signatures in childhood acute leukemias are largely unique and distinct from those of normal tissues and other malignancies
Source: BMC Med Genomics. 2010 Mar 8;3:6. doi: 10.1186/1755-8794-3-6 (PMC2845086; doi:10.1186/1755-8794-3-6)
Supplement: Additional file 10 — Supporting Methods. Contains additional methods information. [file 1755-8794-3-6-S10.DOC]

*SUPPORTING METHODS*

**Gene expression profiling**

RNA extraction was performed as previously described [1]. The quality of total and amplified RNA was assessed by the Agilent 2100 BioAnalyzer (Agilent Technologies, Palo Alto, CA). RNA (150 ng) were linearly amplified using MessageAmp™ II-Biotin *Enhanced* Single Round aRNA Amplification (Ambion, Applied Biosystems, Stockholm, Sweden) in the presence of Affymetrix internal controls. The quality of the amplified cRNA was assessed as above. Fragmentation was performed as described by Affymetrix and 10 µg was used for hybridization onto Human Genome U133 Plus 2.0 microarrays, as described in Methods. Data have been deposited in Gene Expression Omnibus (Accession No GSE19599).

**External data sets** The ALL data set [2] consisted of 118 childhood leukemias, including 20 cases with t(12;21)(p13;q22) [*ETV6/RUNX1*], 18 cases with high hyperdiploidy (>50 chromosomes), 15 cases with t(9;22)(q34;q22)[*BCR/ABL1*], 18 cases with t(1;19)(q23;p13)[*TCF3/PBX1*], 20 cases with 11q23/*MLL*-rearrangements, and 25 cases with a normal karyotype or unclassified genetic changes (designated “others”). The AML data set [3] consisted of 130 AMLs, including 14 with inv(16)(p13;q22)[*CBFB/MYH11*], 15 with t(15;17)(q22;q21)[*PML/RARA*], 21 with t(8;21)(q22;q22)[*RUNX1/RUNX1T1*], 23 with 11q23/*MLL*-rearrangements, 10 with AML M7, and 47 with a normal karyotype or unclassified genetic changes (designated “others”).

**References**

1. Andersson A, Ritz C, Lindgren D, Eden P, Lassen C, Heldrup J, Olofsson T, Rade J, Fontes M, Porwit-Macdonald A, Behrendtz M, Hoglund M, Johansson B, Fioretos T: **Microarray-based classification of a consecutive series of 121 childhood acute leukemias: prediction of leukemic and genetic subtype as well as of minimal residual disease status**. *Leukemia* 2007, **21**(6):1198-1203.

2. Ross ME, Zhou X, Song G, Shurtleff SA, Girtman K, Williams WK, Liu HC, Mahfouz R, Raimondi SC, Lenny N, Patel A, Downing JR: **Classification of pediatric acute lymphoblastic leukemia by gene expression profiling**. *Blood* 2003, **102**(8):2951-2959.

3. Ross ME, Mahfouz R, Onciu M, Liu HC, Zhou X, Song G, Shurtleff SA, Pounds S, Cheng C, Ma J, Ribeiro RC, Rubnitz JE, Girtman K, Williams WK, Raimondi SC, Liang DC, Shih LY, Pui CH, Downing JR: **Gene expression profiling of pediatric acute myelogenous leukemia**. *Blood* 2004, **104**(12):3679-3687.
