# Supplementary material for: Gene expression signatures in childhood acute leukemias are largely unique and distinct from those of normal tissues and other malignancies
Source: BMC Med Genomics. 2010 Mar 8;3:6. doi: 10.1186/1755-8794-3-6 (PMC2845086; doi:10.1186/1755-8794-3-6)
Supplement: Additional file 11 — The normal tissue data set by Su el al., 2005 and the annotations used for producing the gene sets. Cel files and annotations for the data set by Su et al., 2005. [file 1755-8794-3-6-S11.DOC]

**Additional file 11.** The normal tissue data set by Su el al., 2005 [1] and the annotations used for producing the gene sets.

| *CASE NO* | *ORIGINAL ANNOTATION* | *ANNOTATION IN ARTICLE* | *CEL-FILE* |
| --- | --- | --- | --- |
| 1 | Adipocyte | Adipocyte | GSM18975.CEL |
| 2 | Adipocyte | Adipocyte | GSM18976.CEL |
| 3 | Adrenal cortex | Adrenal | GSM18995.CEL |
| 4 | Adrenal cortex | Adrenal | GSM18996.CEL |
| 5 | Adrenal gland | Adrenal | GSM18947.CEL |
| 6 | Adrenal gland | Adrenal | GSM18948.CEL |
| 7 | Appendix | Appendix | GSM18999.CEL |
| 8 | Appendix | Appendix | GSM19000.CEL |
| 9 | Amygdala | Brain | GSM18927.CEL |
| 10 | Amygdala | Brain | GSM18928.CEL |
| 11 | Caudate nucleus | Brain | GSM18919.CEL |
| 12 | Caudate nucleus | Brain | GSM18920.CEL |
| 13 | Cerebellum | Brain | GSM18917.CEL |
| 14 | Cerebellum | Brain | GSM18918.CEL |
| 15 | Cerebellum peduncles | Brain | GSM18915.CEL |
| 16 | Cerebellum peduncles | Brain | GSM18916.CEL |
| 17 | Cingulate cortex | Brain | GSM18939.CEL |
| 18 | Cingulate cortex | Brain | GSM18940.CEL |
| 19 | Fetal brain | Brain | GSM18945.CEL |
| 20 | Fetal brain | Brain | GSM18946.CEL |
| 21 | Globuspallidus | Brain | GSM18913.CEL |
| 22 | Globuspallidus | Brain | GSM18914.CEL |
| 23 | Hypothalamus | Brain | GSM18933.CEL |
| 24 | Hypothalamus | Brain | GSM18934.CEL |
| 25 | Medulla oblongata | Brain | GSM18925.CEL |
| 26 | Medulla oblongata | Brain | GSM18926.CEL |
| 27 | Occipital lobe | Brain | GSM18931.CEL |
| 28 | Occipital lobe | Brain | GSM18932.CEL |
| 29 | Olfactory bulb | Brain | GSM19019.CEL |
| 30 | Olfactory bulb | Brain | GSM19020.CEL |
| 31 | Parietal lobe | Brain | GSM18923.CEL |
| 32 | Parietal lobe | Brain | GSM18924.CEL |
| 33 | Pituitary | Brain | GSM19021.CEL |
| 34 | Pituitary | Brain | GSM19022.CEL |
| 35 | Pons | Brain | GSM18941.CEL |
| 36 | Pons | Brain | GSM18942.CEL |
| 37 | Prefrontal cortex | Brain | GSM18929.CEL |
| 38 | Prefrontal cortex | Brain | GSM18930.CEL |
| 39 | Spinal cord | Brain | GSM18943.CEL |
| 40 | Spinal cord | Brain | GSM18944.CEL |
| 41 | Subthalamic nucleus | Brain | GSM18937.CEL |
| 42 | Subthalamic nucleus | Brain | GSM18938.CEL |
| 43 | Temporal lobe | Brain | GSM18911.CEL |
| 44 | Temporal lobe | Brain | GSM18912.CEL |
| 45 | Thalamus | Brain | GSM18935.CEL |
| 46 | Thalamus | Brain | GSM18936.CEL |
| 47 | Whole brain | Brain | GSM18921.CEL |
| 48 | Whole brain | Brain | GSM18922.CEL |
| 49 | Bronchialepithelial cells | Bronchialepithelial cells | GSM18973.CEL |
| 50 | Bronchialepithelial cells | Bronchialepithelial cells | GSM18974.CEL |
| 51 | Leukemia chronic myelogenous K562 | Cell line | GSM18897.CEL |
| 52 | Leukemia chronic myelogenous K562 | Cell line | GSM18898.CEL |
| 53 | Leukemia lymphoblastic Molt4 | Cell line | GSM18887.CEL |
| 54 | Leukemia lymphoblastic Molt4 | Cell line | GSM18888.CEL |
| 55 | Leukemia promyelocytic HL60 | Cell line | GSM18893.CEL |
| 56 | Leukemia promyelocytic HL60 | Cell line | GSM18894.CEL |
| 57 | Lymphoma Burkitts daudi | Cell line | GSM18895.CEL |
| 58 | Lymphoma Burkitts daudi | Cell line | GSM18896.CEL |
| 59 | Lymphoma Burkitts raji | Cell line | GSM18891.CEL |
| 60 | Lymphoma Burkitts raji | Cell line | GSM18892.CEL |
| 61 | Colorectal adenocarcinoma | Colon cancer(cell line) | GSM18865.CEL |
| 62 | Colorectal adenocarcinoma | Colon cancer(cell line) | GSM18866.CEL |
| 63 | Cardiac myocytes | Heart | GSM18969.CEL |
| 64 | Cardiac myocytes | Heart | GSM18970.CEL |
| 65 | Heart | Heart | GSM18951.CEL |
| 66 | Heart | Heart | GSM18952.CEL |
| 67 | Smooth muscle | Heart | GSM18971.CEL |
| 68 | Smooth muscle | Heart | GSM18972.CEL |
| 69 | 721_B_lymphoblasts | Hematopietic(cell line) | GSM18889.CEL |
| 70 | 721_B_lymphoblasts | Hematopietic(cell line) | GSM18890.CEL |
| 71 | BM-CD105+endothelial | Hematopietic | GSM18883.CEL |
| 72 | BM-CD105+endothelial | Hematopietic | GSM18884.CEL |
| 73 | BM-CD33+myeloid | Hematopietic | GSM18869.CEL |
| 74 | BM-CD33+myeloid | Hematopietic | GSM18870.CEL |
| 75 | BM-CD34+ | Hematopietic | GSM18885.CEL |
| 76 | BM-CD34+ | Hematopietic | GSM18886.CEL |
| 77 | BM-CD71+early erythroid | Hematopietic | GSM18907.CEL |
| 78 | BM-CD71+early erythroid | Hematopietic | GSM18908.CEL |
| 79 | PB-bdca4+dentritic cells | Hematopietic | GSM18873.CEL |
| 80 | PB-bdca4+dentritic cells | Hematopietic | GSM18874.CEL |
| 81 | PB-CD14+monocytes | Hematopietic | GSM18871.CEL |
| 82 | PB-CD14+monocytes | Hematopietic | GSM18872.CEL |
| 83 | PB-CD19+B cells | Hematopietic | GSM18881.CEL |
| 84 | PB-CD19+B cells | Hematopietic | GSM18882.CEL |
| 85 | PB-CD4+T cells | Hematopietic | GSM18877.CEL |
| 86 | PB-CD4+T cells | Hematopietic | GSM18878.CEL |
| 87 | PB-CD56+NK cells | Hematopietic | GSM18875.CEL |
| 88 | PB-CD56+NK cells | Hematopietic | GSM18876.CEL |
| 89 | PB-CD8+tcells | Hematopietic | GSM18879.CEL |
| 90 | PB-CD8+tcells | Hematopietic | GSM18880.CEL |
| 91 | Whole blood | Hematopietic | GSM18867.CEL |
| 92 | Whole blood | Hematopietic | GSM18868.CEL |
| 93 | Kidney | Kidney | GSM18955.CEL |
| 94 | Kidney | Kidney | GSM18956.CEL |
| 95 | Fetal liver | Liver | GSM18905.CEL |
| 96 | Fetal liver | Liver | GSM18906.CEL |
| 97 | Liver | Liver | GSM18953.CEL |
| 98 | Liver | Liver | GSM18954.CEL |
| 99 | Fetal lung | Lung | GSM18965.CEL |
| 100 | Fetal lung | Lung | GSM18966.CEL |
| 101 | Lung | Lung | GSM18949.CEL |
| 102 | Lung | Lung | GSM18950.CEL |
| 103 | Bone marrow | Lymphatic tissues | GSM18909.CEL |
| 104 | Bone marrow | Lymphatic tissues | GSM18910.CEL |
| 105 | Lymphnode | Lymphatic tissues | GSM18903.CEL |
| 106 | Lymphnode | Lymphatic tissues | GSM18904.CEL |
| 107 | Thymus | Lymphatic tissues | GSM18899.CEL |
| 108 | Thymus | Lymphatic tissues | GSM18900.CEL |
| 109 | Tonsil | Lymphatic tissues | GSM18901.CEL |
| 110 | Tonsil | Lymphatic tissues | GSM18902.CEL |
| 111 | Skeletal muscle | Muscle | GSM19013.CEL |
| 112 | Skeletal muscle | Muscle | GSM19014.CEL |
| 113 | Tongue | Muscle | GSM19017.CEL |
| 114 | Tongue | Muscle | GSM19018.CEL |
| 115 | Atrioventricular node | Neural | GSM19007.CEL |
| 116 | Atrioventricular node | Neural | GSM19008.CEL |
| 117 | Ciliary ganglion | Neural | GSM19003.CEL |
| 118 | Ciliary ganglion | Neural | GSM19004.CEL |
| 119 | Drg | Neural | GSM19009.CEL |
| 120 | Drg | Neural | GSM19010.CEL |
| 121 | Superior cervical ganglion | Neural | GSM19011.CEL |
| 122 | Superior cervical ganglion | Neural | GSM19012.CEL |
| 123 | Trigeminal ganglion | Neural | GSM19005.CEL |
| 124 | Trigeminal ganglion | Neural | GSM19006.CEL |
| 125 | Ovary | Ovary | GSM18997.CEL |
| 126 | Ovary | Ovary | GSM18998.CEL |
| 127 | Pancreas | Pancreas | GSM18977.CEL |
| 128 | Pancreas | Pancreas | GSM18978.CEL |
| 129 | Pancreatic islets | Pancreas | GSM18979.CEL |
| 130 | Pancreatic islets | Pancreas | GSM18980.CEL |
| 131 | Placenta | Placenta | GSM18967.CEL |
| 132 | Placenta | Placenta | GSM18968.CEL |
| 133 | Prostate | Prostate | GSM18957.CEL |
| 134 | Prostate | Prostate | GSM18958.CEL |
| 135 | Salivary gland | Salivary gland | GSM18991.CEL |
| 136 | Salivary gland | Salivary gland | GSM18992.CEL |
| 137 | Skin | Skin | GSM19001.CEL |
| 138 | Skin | Skin | GSM19002.CEL |
| 139 | Testis | Testis | GSM18981.CEL |
| 140 | Testis | Testis | GSM18982.CEL |
| 141 | Testis germ cell | Testis | GSM18985.CEL |
| 142 | Testis germ cell | Testis | GSM18986.CEL |
| 143 | Testis interstitial | Testis | GSM18987.CEL |
| 144 | Testis interstitial | Testis | GSM18988.CEL |
| 145 | Testis leydig cell | Testis | GSM18983.CEL |
| 146 | Testis leydig cell | Testis | GSM18984.CEL |
| 147 | Testis seminiferous tubule | Testis | GSM18989.CEL |
| 148 | Testis seminiferous tubule | Testis | GSM18990.CEL |
| 149 | Trachea | Trachea | GSM18993.CEL |
| 150 | Trachea | Trachea | GSM18994.CEL |
| 151 | Fetal thyroid | Tyroid | GSM18963.CEL |
| 152 | Fetal thyroid | Tyroid | GSM18964.CEL |
| 153 | Thyroid | Tyroid | GSM18961.CEL |
| 154 | Thyroid | Tyroid | GSM18962.CEL |
| 155 | Uterus | Uterus | GSM18959.CEL |
| 156 | Uterus | Uterus | GSM18960.CEL |
| 157 | Uterus corpus | Uterus Corpus | GSM19015.CEL |
| 158 | Uterus corpus | Uterus Corpus | GSM19016.CEL |

1. Su AI, Wiltshire T, Batalov S, Lapp H, Ching KA, Block D, Zhang J, Soden R, Hayakawa M, Kreiman G, Cooke MP, Walker JR, Hogenesch JB: **A gene atlas of the mouse and human protein-encoding transcriptomes**. *Proc Natl Acad Sci U S A* 2004, **101**(16):6062-6067.
