# Supplementary material for: Gene expression signatures in childhood acute leukemias are largely unique and distinct from those of normal tissues and other malignancies
Source: BMC Med Genomics. 2010 Mar 8;3:6. doi: 10.1186/1755-8794-3-6 (PMC2845086; doi:10.1186/1755-8794-3-6)
Supplement: Additional file 12 — The solid tumor data set from the expO database. Description of the analyzed tumors. [file 1755-8794-3-6-S12.DOC]

**Additional file 12. The solid tumor data set from the expO database.**

| *CASE* | *SITE* | *HISTOLOGY* | *CEL FILE* |
| --- | --- | --- | --- |
| 1 | Brain | - | GSM117587 |
| 2 | Brain | - | GSM117769 |
| 3 | Brain | - | GSM76588 |
| 4 | Breast | Ductal Carcinoma | GSM38106 |
| 5 | Breast | Ductal Carcinoma | GSM38110 |
| 6 | Breast | Ductal Carcinoma | GSM46855 |
| 7 | Breast | Ductal Carcinoma | GSM46874 |
| 8 | Breast | Ductal Carcinoma | GSM46885 |
| 9 | Breast | Ductal Carcinoma | GSM46890 |
| 10 | Breast | Ductal Carcinoma | GSM46893 |
| 11 | Colon | Adenocarcinoma | GSM53045 |
| 12 | Colon | Adenocarcinoma | GSM53073 |
| 13 | Colon | Adenocarcinoma | GSM53113 |
| 14 | Colon | Adenocarcinoma | GSM53142 |
| 15 | Colon | Adenocarcinoma | GSM53148 |
| 16 | Colon | Adenocarcinoma | GSM53156 |
| 17 | Colon | Adenocarcinoma | GSM88999 |
| 18 | Endometrium | Endometrioid carcinoma | GSM46854 |
| 19 | Endometrium | Endometrioid carcinoma | GSM46912 |
| 20 | Endometrium | Endometrioid carcinoma | GSM46914 |
| 21 | Endometrium | Endometrioid carcinoma | GSM53143 |
| 22 | Endometrium | Endometrioid carcinoma | GSM53149 |
| 23 | Endometrium | Endometrioid carcinoma | GSM53155 |
| 24 | Endometrium | Endometrioid carcinoma | GSM53164 |
| 25 | Endometrium | Endometrioid carcinoma | GSM76536 |
| 26 | Endometrium | Endometrioid carcinoma | GSM76638 |
| 27 | Exocrine Pancreas | Ductal adenocarcinoma | GSM117645 |
| 28 | Exocrine Pancreas | Ductal adenocarcinoma | GSM137958 |
| 29 | Exocrine Pancreas | Ductal adenocarcinoma | GSM89045 |
| 30 | Kidney | Conventional (clear cell) renal carcinoma | GSM46944 |
| 31 | Kidney | Conventional (clear cell) renal carcinoma | GSM46963 |
| 32 | Kidney | Conventional (clear cell) renal carcinoma | GSM46965 |
| 33 | Kidney | Conventional (clear cell) renal carcinoma | GSM53043 |
| 34 | Kidney | Conventional (clear cell) renal carcinoma | GSM53064 |
| 35 | Lung | Adenocarcinoma, NOS | GSM46941 |
| 36 | Lung | Adenocarcinoma, NOS | GSM88962 |
| 37 | Lung | Squamous cell carcinoma | GSM117629 |
| 38 | Lung | Squamous cell carcinoma | GSM46850 |
| 39 | Lung | Squamous cell carcinoma | GSM46868 |
| 40 | Lung | Squamous cell carcinoma | GSM46936 |
| 41 | Lung | Squamous cell carcinoma | GSM46973 |
| 42 | Lung | Squamous cell carcinoma | GSM53167 |
| 43 | Lymph Nodes (Nodal Lymphoma) | Follicular lymphoma | GSM53038 |
| 44 | Lymph Nodes (Nodal Lymphoma) | Follicular lymphoma | GSM53101 |
| 45 | Ovary | Endometrioid Carcinoma | GSM46830 |
| 46 | Ovary | Metastatic Papillary Serous Carcinoma | GSM38065 |
| 47 | Ovary | Metastatic Papillary Serous Carcinoma | GSM38066 |
| 48 | Ovary | Papillary Serous Carcinoma | GSM38064 |
| 49 | Ovary | Papillary Serous Carcinoma | GSM38088 |
| 50 | Ovary | Papillary Serous Carcinoma | GSM46966 |
| 51 | Ovary | Papillary Serous Carcinoma | GSM53150 |
| 52 | Ovary | Papillary Serous Carcinoma | GSM53163 |
| 53 | Prostate | Adenocarcinoma, Acinar Type | GSM53162 |
| 54 | Prostate | Adenocarcinoma, Acinar Type | GSM76553 |
| 55 | Prostate | Adenocarcinoma, Acinar Type | GSM76640 |
| 56 | Prostate | Adenocarcinoma, Acinar Type | GSM89017 |
| 57 | Prostate | Adenocarcinoma, NOS | GSM53114 |
| 58 | Prostate | Adenocarcinoma, NOS | GSM76516 |
| 59 | Prostate | Adenocarcinoma, NOS | GSM76648 |
| 60 | Prostate | Adenocarcinoma, NOS | GSM88977 |
| 61 | Soft Tissue Lt Thigh | Myxoid pleomorphic spindle cell sarcoma | GSM89088 |
| 62 | Soft tissue of R Thigh | Synovial sarcoma | GSM137930 |
| 63 | Soft Tissue of thigh | Myxoid liposarcoma | GSM117732 |
| 64 | Soft Tissue Sarcoma | Pleomorphic sarcoma | GSM102532 |
| 65 | Thyroid | Papillary carcinoma | GSM138023 |
| 66 | Thyroid | Papillary carcinoma | GSM138024 |
| 67 | Thyroid | Papillary carcinoma | GSM88998 |
| 68 | Thyroid | Papillary carcinoma | GSM89038 |
| 69 | Thyroid | Papillary carcinoma, follicular variant | GSM102424 |
| 70 | Thyroid | Papillary carcinoma, follicular variant | GSM117705 |
| 71 | Thyroid | Papillary carcinoma, follicular variant | GSM89032 |
| 72 | Urinary Bladder | Urothelial (transitional cell) carcinoma | GSM102437 |
| 73 | Urinary Bladder | Urothelial (transitional cell) carcinoma | GSM137919 |
| 74 | Urinary Bladder | Urothelial (transitional cell) carcinoma | GSM53117 |
| 75 | Urinary Bladder | Urothelial (transitional cell) carcinoma | GSM88993 |
